# C–H and C–F bond activation of fluorinated propenes at Rh: enabling cross-coupling reactions with outer-sphere C–C coupling†‡

**DOI:** 10.1039/d4sc00951g

**Published:** 2024-05-02

**Authors:** Maria Talavera, Soodeh Mollasalehi, Thomas Braun

**Affiliations:** a Facultad de Química, Universidade de Vigo Campus Universitario 36310 Vigo Spain matalaveran@uvigo.gal; b Department of Chemistry, Humboldt Universität zu Berlin Brook-Taylor Straße 2 12489 Berlin Germany thomas.braun@cms.hu-berlin.de

## Abstract

The reaction of [Rh{(*E*)-CF=CHCF_3_}(PEt_3_)_3_] with Zn(CH_3_)_2_ results in the methylation of the alkenyl ligand to give [Rh{(*E*/*Z*)-C(CH_3_)=CHCF_3_}(PEt_3_)_3_]. Variable temperature NMR studies allowed the identification of a heterobinuclear rhodium–zinc complex as an intermediate, for which the structure [Rh(CH_3_)(ZnCH_3_){(*Z*)-C(CH_3_)=CHCF_3_}(PEt_3_)_2_] is proposed. Based on these stoichiometric reactions, unique Negishi-type catalytic cross-coupling reactions of fluorinated propenes by consecutive C–H and C–F bond activation steps at room temperature were developed. The C–H bond activation steps provide a fluorinated ligand at Rh and deliver the fluorinated product, whereas the C–F bond activation and C–C coupling occur *via* outer-sphere nucleophilic attack at the fluorinated alkenyl ligand.

## Abstract 2

Cross coupling reactions of fluorinated olefins occur by C–H bond activation at Rh to provide a fluorinated ligand for a subsequent outer-sphere C–F bond functionalization.

## Graphical abstract

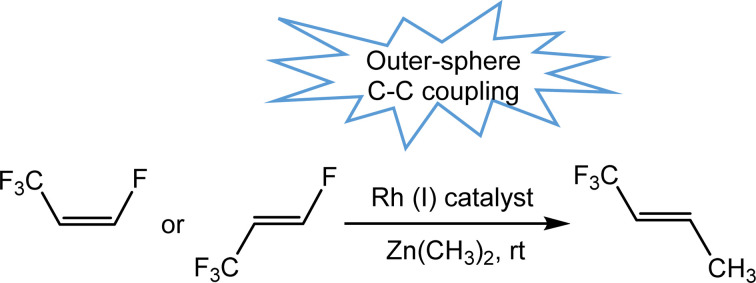

## Introduction

Fluorinated compounds are of eminent importance as they are used in a broad range of fields such as pharmaceutics, agrochemicals and materials science.^1–4^ Fluorinated olefins are currently indispensable, because they have applications in automobile air-conditioning systems and as monomers for polymeric compounds such as Teflon.^5–7^ While the synthesis of fluorinated olefins has been widely developed through fluorination reactions,^8,9^ the activation and functionalization of olefinic or vinylic C(sp^2^)–F bonds is less common,^10–13^ and often it is mediated by a transition metal complex.^12,13^

A possible functionalization pathway would consist of the formation of C–C bonds by cross-coupling reactions. Such conversions have been investigated for chlorinated or brominated olefin derivatives.^14,15^ For fluoroalkenes, C–C couplings have been less studied and examples include group 10 catalysts and the use of *gem*-difluoroalkenes.^16–28^ The couplings proceed by an initial C–F bond oxidative addition – in some cases using lithium salts to promote the activation step – or insertion of a fluorinated olefin into a metal–carbon bond followed by β-fluorine elimination. Cao and Wu developed a Suzuki–Miyaura cross coupling arylation of *gem*-difluoroalkenes using a nickel catalyst,^21^ whereas Tsui and Liu applied a palladium catalyst.^16^ Xu *et al.* published the arylation of an *in situ* formed difluorovinyl ketone in the presence of a palladium catalyst and aryl boronic acids.^19^ Regarding rhodium catalyzed reactions, Xia and co-workers described the formation of monofluorinated dienes from *gem*-difluorocyclopropanes, which are employed as fluoroallyl surrogates, by C–C coupling with allylboronates.^29^ Negishi-type cross-coupling reactions based on vinyl C(sp^2^)–F bond oxidative addition steps are rare. Saeki *et al.* functionalized the olefin CF_2_=CH(1-naphtyl) with arylzinc derivative MeC_6_H_4_ZnCl in the presence of a palladium catalyst.^30^ In addition, examples using Pd or Ni catalysts and lithium salts to promote the C–F bond activation have been described by Zhang *et al.* and Ogoshi and co-workers developing Negishi-type C(sp^2^)–F bond alkylations of 2,3,3,3-tetrafluoropropene or tetrafluoroethylene, respectively.^31,32^

**Scheme 1:**
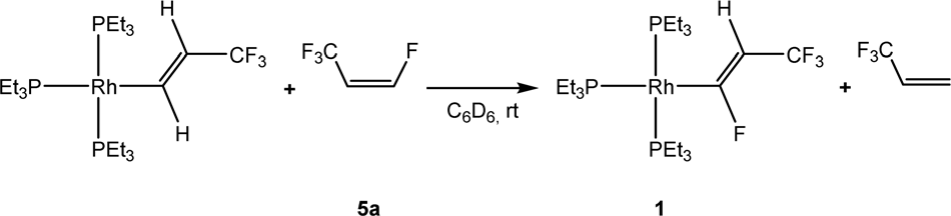
Reaction of [Rh{(*E*)-CH=CHCF_3_}(PEt_3_)_3_] with *Z*-1,3,3,3-tetrafluoropropene.^33^

Recently, the reactivity of *Z*-1,3,3,3-tetrafluoropropene (5a) towards rhodium(i) complexes has been studied and it was shown that rhodium alkenyl complexes with a low fluoroorganyl content such as [Rh{(*E*)-CH=CHCF_3_}(PEt_3_)_3_] can activate higher fluorinated olefins (Scheme 1). For the latter, in a C–H activation step [Rh{(*E*)-CF=CHCF_3_}(PEt_3_)_3_] (1) and 3,3,3-trifluoropropene were produced.^33^

Herein studies on catalytic C–C coupling reactions at fluorinated alkenyl ligands are described. Stoichiometric model reactions give an insight into a possible reaction mechanism. Unprecedented rhodium catalyzed Negishi-type cross-coupling reactions of fluorinated olefins have been developed, based on both C(sp^2^)–F and C(sp^2^)–H bond activation and, remarkably, involving a C–C coupling step in the outer coordination sphere.

## Results and discussion

**Scheme 2:**
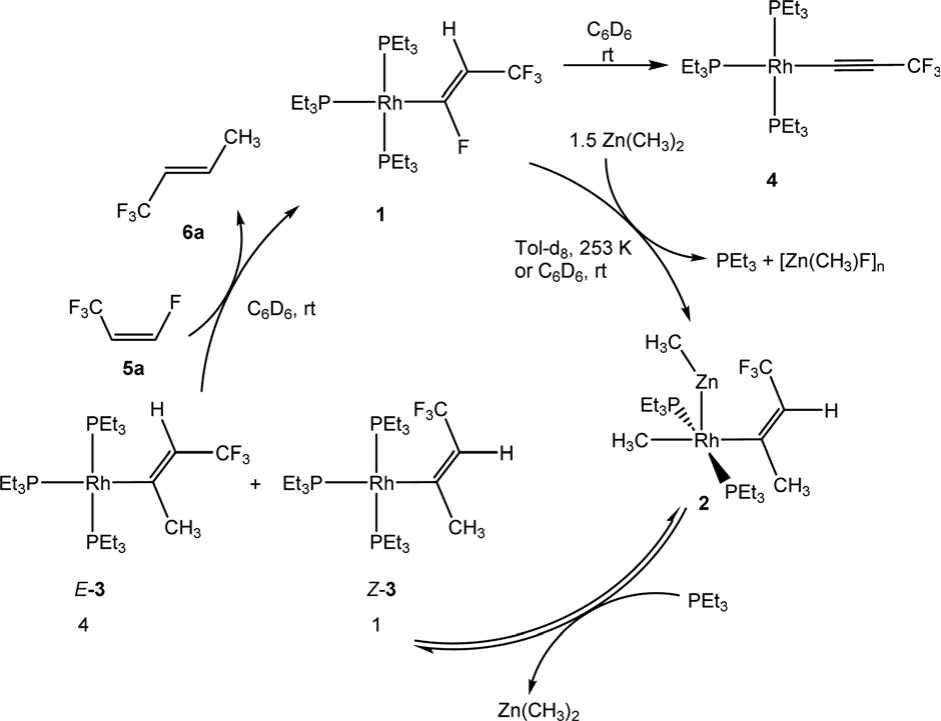
Reactivity of complex 1 towards Zn(CH_3_)_2_.

Treatment of complex [Rh{(*E*)-CF=CHCF_3_}(PEt_3_)_3_] (1) with one equivalent of Zn(CH_3_)_2_ in C_6_D_6_ yielded after 20 min at room temperature a mixture of complexes, which included a heterobimetallic compound, for which the structure [Rh(CH_3_)(ZnCH_3_){(*Z*)-C(CH_3_)=CHCF_3_}(PEt_3_)_2_] (2) is suggested, as well as the isomers [Rh{(*E*)-C(CH_3_)=CHCF_3_}(PEt_3_)_3_] (*E*-3), [Rh{(*Z*)-C(CH_3_)=CHCF_3_}(PEt_3_)_3_] (*Z*-3), the alkynyl complex [Rh(C≡CCF_3_)(PEt_3_)_3_] (4) and an unknown product in a ratio of 3 : 0.7 : 2.5 : 1, respectively. When the reaction was run in the presence of one equivalent of PEt_3_, a mixture of complexes 2 and *Z*-3 in a 1 : 17 ratio was observed after 10 minutes. The preferential formation of the *Z*-3 isomer might indicate a diminished role of 2 for the reaction mechanism in this case. When the reaction was monitored at 253 K by NMR spectroscopy, after 4 h 75% conversion of complex 1 was observed to give complex 2 and a second complex (ratio 9 : 1), which is possibly an isomer of 2, together with the release of triethylphosphine (Scheme 2). The rhodium-zinc heterobimetallic complex 2 is not stable at low temperatures, and after one day, reductive elimination of Zn(CH_3_)_2_ took place followed by coordination of phosphine to rhodium leading to the *Z*/*E*-3 isomers in a 1 : 4 ratio. Treatment of the product mixture with *Z*-1,3,3,3-tetrafluoropropene (5a) led then to the regeneration of complex 1 and release of *E*-1,1,1-trifluorobut-2-ene (6a) by C–H bond activation and formation (Scheme 2). This step comprises the replacement of a lower fluorinated ligand by a higher fluorinated ligand by C–H bond activation as a higher degree of fluorination often leads to stronger bonds.^34^ As previously reported, complex 1 is not stable and after a dehydrofluorination step complex 4 is obtained.^33^

In an alternative approach in which complex 1 was treated with LiCH_3_ instead of Zn(CH_3_)_2_, a mixture of complexes (*Z*/*E*)-3 and 4 was also obtained. However, in this case a heterobinuclear intermediate similar to 2 was not observed. Note that Rh–Zn bimetallic complexes have been described before.^35–39^

The formation of 2 followed by the generation of *E*/*Z*-3 from complex 1 can be considered as a case for a rare outer-sphere reaction where a nucleophile attacks the fluorinated ligand bonded at rhodium. Note that the attack of a fluorosilicate at the carbon atom of a fluorinated pyridyl ligand has been proposed at rhodium.^40^ The attack of PEt_3_ at the β-carbon atom of a perfluorovinyl ligand at nickel was also described.^41^ In addition, outer-sphere electrophilic fluorination was reported by Lynam, Slattery and co-workers.^42,43^ The rearrangements of the alkenyl ligands for the conversion of 2 into *E*-3, 1 into 2 and *E*-3 into 6a comprise an isomerization at the double bond. This might involve an intermediate vinylidene complex that can be formed by a reversible migration of a methyl group from the alpha carbon to the metal center. Intermediate metallacyclopropene-like species have also been discussed for such rearrangements.^44–46^ In addition, a negative hyperconjugation of the π-electron density into antibonding orbitals at the CF_3_ group might weaken the C=C double bond and allow for an isomerization of the alkenyl ligands. Note that Ojima *et al.* proposed a zwitterionic carbene–rhodium complex as an intermediate for such a *cis*/*trans* rearrangement.^47^ However, another alternative route for the isomerization to give complexes 2 or *E*-3 could imply a rearrangement during the nucleophilic substitution. After addition of CH_3_^−^ to Cα, the β-carbanion formed could show a free rotation about Cα–Cβ. A Lewis-acidic cation of the type [ZnMe]^+^ would then abstract the fluoride forming the oligomeric [ZnFMe]_*n*_.

The suggested structure of complex 2 is supported by NMR spectroscopic data, in part based on the data for the ^13^C labeled derivative [Rh(^13^CH_3_)(Zn^13^CH_3_){(*Z*)-C(^13^CH_3_)=CHCF_3_}(PEt_3_)_2_] (2′) (see ESI‡). Thus, in the ^1^H NMR spectrum of complex 2′ a resonance for the rhodium-bound methyl ligand is observed at −0.42 ppm as a doublet of triplets with coupling constants of 121.1 and 4.6 Hz due to the coupling to the carbon atom and the two phosphine ligands. In addition, a resonance at −0.21 ppm is assigned to the methyl group at the Zn atom. It appears as a doublet coupled with ^1^*J*_H–C_ = 123.2 Hz. The ^13^C{^1^H} NMR displays two resonances at −27.5 and −9.2 ppm for the rhodium- and zinc-bonded methyl moieties, respectively. The former exhibits a doublet of pseudo quartets as a result of the coupling to rhodium (9.3 Hz), the two phosphorus atoms and the carbon atom bonded to zinc with a coupling constant of 5.2 Hz. The latter signal also shows the 5.2 Hz carbon–carbon coupling together with the extra coupling to rhodium resulting in a pseudo triplet. In addition, the geometry of complex 2 was optimized by DFT calculations using toluene as a solvent (BP86/def2-SVP, see ESI‡). The complex exhibits a tetragonal pyramidal structure at rhodium in which the methylzinc group is at the apical position. The distance between the Zn nucleus and one of the fluorine atoms of the CF_3_ moiety of 2.747 Å is shorter than the sum of van der Waals radii which might be the reason for the favored *cis* arrangement of the Zn center and the CF_3_ group at the moiety containing the double bond. In fact, this product is 7.4 kJ mol^−1^ more stable than the corresponding *trans* isomer.

**Scheme 3:**
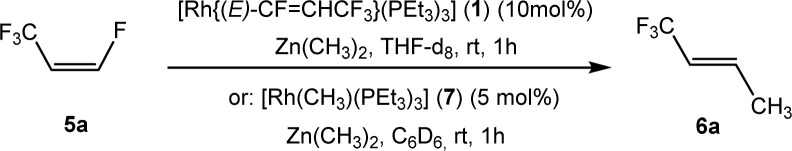
Catalytic Negishi cross-coupling reaction of 5a.

Based on the stoichiometric functionalization of the fluorinated alkenyl ligand at complex 1 to yield 6a, a process for the catalytic methylation of fluoroolefins was developed. The reaction of *Z*-1,3,3,3-tetrafluoropropene (5a) with Zn(CH_3_)_2_ in THF-d_8_ in the presence of 10 mol% [Rh{(*E*)-CF=CHCF_3_}(PEt_3_)_3_] (1) as a catalyst gave full conversion of the olefin into the C–C coupling product *E*-1,1,1-trifluorobut-2-ene (6a) (Scheme 3, Table 1, entry 1).

**Table 1:** Catalyst and methyl source screening to obtain 6a

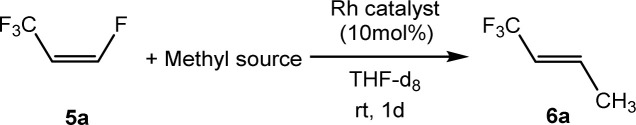			
Entry	Catalyst	Methyl source	Conversion^a^ (%)
1	Complex 1	Zn(CH_3_)_2_	99
2^b^	Complex 7	Zn(CH_3_)_2_	99
3	[Rh(F)(PEt_3_)_3_]	Zn(CH_3_)_2_	70
4	[Rh(H)(PEt_3_)_3_]	Zn(CH_3_)_2_	70
5	Complex 7	MgBrCH_3_	—
6	Complex 7	LiCH_3_	—
7	Complex 7	Zn(CH_3_)Cl	15

a Conversion based on the consumption of Zn(CH_3_)_2_ and determined by ^1^H NMR spectroscopy.

b 1 h reaction time, 5 mol% of complex 7.

Complex [Rh{(*E*)-CF=CHCF_3_}(PEt_3_)_3_] (1) is synthesized by C–H bond activation of 5a using [Rh(CH_3_)(PEt_3_)_3_] (7).^33^ Therefore, complex 7 was also tested as a pre-catalyst and full conversion to compound 6a was obtained after 1 h using only 5 mol% of catalyst (Scheme 3, Table 1, entry 2). Remarkably the transformation occurs at room temperature within 1 h. Note that Zn(CH_3_)_2_ does not react with the olefin in the absence of a rhodium catalyst.

In general, there are few examples of rhodium-mediated Negishi-type cross-coupling reactions described in the literature. For these cases, C–I bond activation functionalization steps do not proceed through an outer-sphere reaction; instead they are proposed to occur *via* common oxidative addition/transmetalation cycles. Thus, Takagi *et al.* described alkyl-aryl C–C coupling reactions using [RhCl(cyclooctadiene)]_2_ with bidentate phosphines^48^ and work by Ozerov and co-workers using a PNP pincer rhodium complex as a precatalyst afforded a 7% yield in an aryl–aryl coupling.^39^ It might additionally be worth noting that examples on the functionalization of fluoroaromatics by cross-coupling reactions involving C–F bond cleavage have been reported, for instance Negishi-type conversions at Ni and Pd by the research groups of Love, Ogoshi and Radius.^13,49–64^

Other rhodium complexes as catalysts for the derivatization of 5a were then studied using Zn(CH_3_)_2_ as a nucleophile. Accordingly, Zn(CH_3_)_2_ methylates the Rh–F bond of [Rh(F)(PEt_3_)_3_] and in the presence of *Z*-1,3,3,3-tetrafluoropropene (5a), the generation of *E*-1,1,1-trifluorobut-2-ene (6a) with 70% conversion was achieved (Table 1, entry 3). A comparable outcome was obtained when [Rh(H)(PEt_3_)_3_] was tested as a catalyst (Table 1, entry 4). Note that the rhodium hydrido complex is known to activate preferentially vinylic C–F bonds followed by C–H bond activation of another equivalent of olefin.^65–68^

As an alternative to Zn(CH_3_)_2_, BrMgCH_3_ and LiCH_3_ were applied for the catalytic methylation of 5a, but the formation of the methylated derivative was not observed within one day (Table 1, entries 5 and 6). Note that an oligomerization of the nucleophilic reagents in solution can attenuate the nucleophilicity of their methyl anion. On the other hand, mono organylzinc chloride derivatives can also be used for cross-coupling reactions. Indeed, chloromethylzinc could be employed to yield 6a, but only with 15% conversion (Table 1, entry 7).

**Scheme 4:**
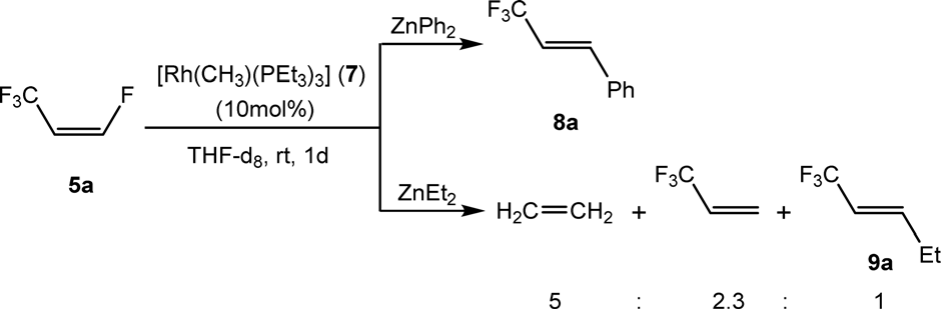
Activation of compound 5a with ZnPh_2_ and ZnEt_2_.

Further functionalization of *Z*-1,3,3,3-tetrafluoropropene (5a) was attempted using ZnEt_2_ and ZnPh_2_ as nucleophile sources. Indeed, reaction of 5a with ZnPh_2_ in the presence of complex 7 as a catalyst gave, within 1 d, *E*-3-phenyl-1,1,1-trifluoropropene (8a) with 90% conversion (Scheme 4), but after one week at room temperature, full conversion was achieved. Interestingly, ZnEt_2_ provided a different outcome. The reaction of 5a with ZnEt_2_ using complex 7 as a catalyst gave, after 1 day a mixture of ethylene, *E*-1,1,1-trifluoropent-2-ene (9a) and 3,3,3-trifluoropropene in a 5 : 1 : 2.3 ratio with 60% conversion (Scheme 4).

The formation of 9a should take place through a comparable mechanism as for the generation of 6a, leading to a Rh–Zn bimetallic intermediate which would bear an ethyl group at the olefin and an ethyl ligand bound to rhodium. This intermediate can produce ethene together with [Rh(H)(PEt_3_)_3_] by β-H elimination. The rhodium hydrido complex would then react with 5a to give 3,3,3-trifluoropropene and complex [Rh{(*E*)-CF=CHCF_3_}(PEt_3_)_3_] (1) as described before in the literature.^33^

The scope of the rhodium defluorinative methylation was then investigated using other olefinic substrates and complex [Rh(CH_3_)(PEt_3_)_3_] (7) as a catalyst (Table 2, see also the ESI‡ for further optimizations). Thus, *E*-1,3,3,3-tetrafluoropropene (5b) was converted into compound 6a with 95% conversion. Higher fluorinated derivatives were also attempted and both *Z*-1,2,3,3,3-pentafluoropropene (5c) and perfluoropropene (5d) were used. In the first case *Z*-1,1,1,2-tetrafluorobut-2-ene (6c) was identified in 25% conversion as the main product, while in the second case a mixture of *Z*/*E*-1,2,3,3,3-pentafluorobut-2-ene isomers (6d) in a 1 : 0.9 ratio was formed (10% conversion). The latter stoichiometric conversion and decrease in reactivity for the perfluorinated substrate could be due to the preference of complex 7 for the activation of C–H over C–F bonds.^67^ Then, 2,3,3,3-tetrafluoropropene (HFO-1234yf, 5e) was used as a substrate, but only 11% conversion was achieved to form 6c and unidentified compounds. Finally, to investigate the influence of a CF_3_ group on the catalytic methylation of fluoroolefins, 1,1-difluoroethane (5f) and trifluoroethane (5g) were employed as reagents. While 5f yielded only traces of the methylated product 2-fluoropropene (6f) even at 60 °C, compound 5g provided a mixture of *E*-1,2-difluoropropene (6g) and an unknown compound in a 5 : 1 ratio with 17% conversion. These results also suggest a preference of the rhodium system for olefins with a geminal CHF group facilitating the initial C–H bond activation.

**Table 2:** Negishi cross-coupling methylation of fluorinated olefins (R^F^ = F and/or CF_3_)

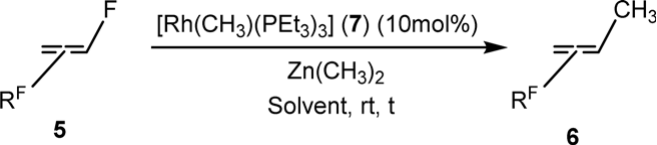				
Olefin	Solvent	Time	Conv.^a^ (%)	Products
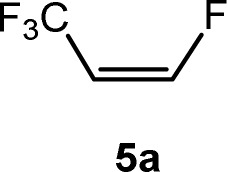	C_6_D_6_	1 h	99	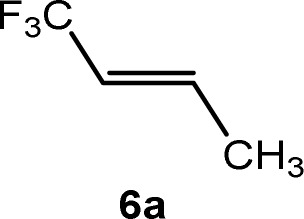
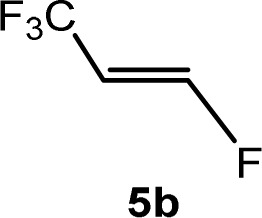	C_6_D_6_	6 d	95	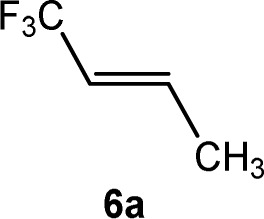
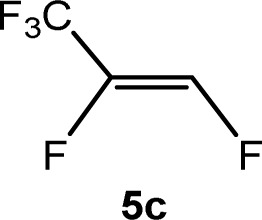	THF-d_8_	1 d	25^c^	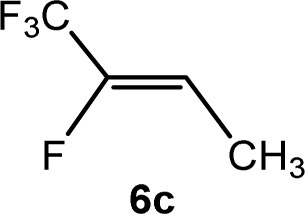
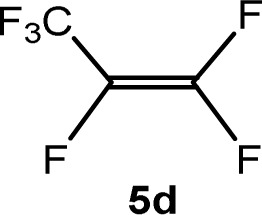	THF-d_8_	1 d	10	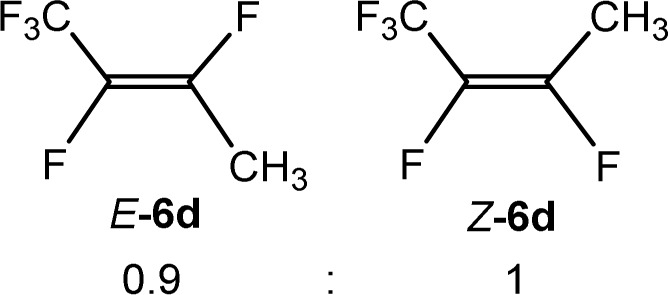
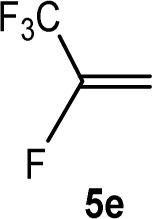	THF-d_8_	6 d	11	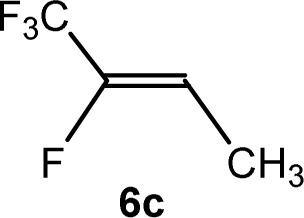
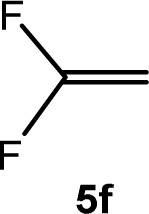	C_6_D_6_	4 d^b^	Traces	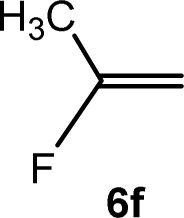
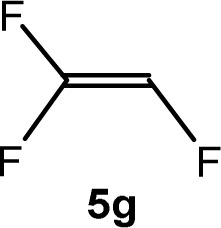	THF-d_8_	1 d	17	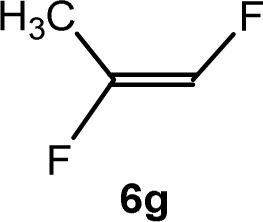

a Determined by ^1^H and ^19^F NMR.

b At 60 °C.

c Main product.

## Conclusions

In conclusion, Negishi-type cross-coupling reactions of fluorinated alkenes were developed. The conversions follow an unprecedented reaction pathway: (I) the C–F bond activation and C–C coupling steps occur by an outer-sphere nucleophilic attack at the fluorinated alkenyl ligand. (II) Another crucial step comprises C–H bond activation to convert the olefinic substrate into a Rh derivative, whereas at the same time the lower-fluorinated olefinic product is released. Stoichiometric model reactions give insight into key-steps of a putative catalytic cycle. A heterobinuclear Rh/Zn complex might play a certain role for the C–C coupling step. Note that in the past Rh-catalyzed hydrodefluorination, germylation, silylation and borylation reactions of fluorinated olefins were studied, but cross coupling reactions were elusive.^65–71^

## Data availability

Details of experimental procedures, characterization of the complexes can be found in the ESI.‡

## Author contributions

Conceptualization, M. T. and T. B.; investigation, M. T., S. M.; writing—original draft preparation, M. T.; writing—review and editing, M. T., S. M. and T. B.; funding acquisition, T. B.

## Conflicts of interest

There are no conflicts to declare.

## Supplementary Material

SC-015-D4SC00951G-s001
