# Supplementary material for: C–H and C–F bond activation of fluorinated propenes at Rh: enabling cross-coupling reactions with outer-sphere C–C coupling
Source: Chem Sci. 2024 May 2;15(22):8472–7. doi: 10.1039/d4sc00951g (PMC11151818; doi:10.1039/d4sc00951g)
Supplement: SC-015-D4SC00951G-s001 [file SC-015-D4SC00951G-s001.pdf]

Electronic Supplementary Material (ESI) for Chemical Science.
This journal is © The Royal Society of Chemistry 2024

Electronic Supporting Information

C−H and C−F bond activation of fluorinated propenes at Rh: enabling cross-coupling reactions by outer-sphere C−C coupling

Maria Talavera*[a,b] Soodeh Mollasalehi[b] and Thomas Braun*[b]

[a] *Facultad de Química, Universidade de Vigo, Campus Universitario, 36310 Vigo (Spain). E-mail: matalaveran@uvigo.gal*

[b] *Department of Chemistry, Humboldt-Universität zu Berlin, Brook-Taylor-Str. 2, 12489 Berlin, Germany. E-mail: thomas.braun@cms.hu-berlin.de.*

Table of contents

1. Synthesis and characterization of all compounds 3

1.1 General Procedures, Methods and Materials 3

1.2 Reaction of [Rh{(*E*)-CF=CHCF$_3$}(PEt$_3$)$_3$] (**1**) with Zn(CH$_3$)$_2$ 3

1.3 General procedure for the catalytic Negishi coupling at fluoroolefins 5

1.4 Optimization attempts 8

1.5 NMR Spectra 9

2. DFT calculations 20

2.1 Computational details for geometry optimization of the calculated complexes 20

2.2 Geometry optimization of complex **2** 20

3. References 23

1. Synthesis and characterization of all compounds

1.1 General Procedures, Methods and Materials

All experiments were carried out under an atmosphere of argon by Schlenk techniques. Solvents were dried by the usual procedures[1] and, prior to use, distilled under argon. The rhodium complexes [Rh(CH_3)(PEt_3)$_3$] (**7**)[2], [Rh{(*E*)-CH=CHCF$_3$}(PEt_3)$_3$] (**1**)[3] and the other rhodium catalyst used[4] as well as the zinc reagents $ZnPh_2$[5] and Zn($^{13}CH_3$)$_2$[6] were prepared as described in the literature. All reagents were obtained from commercial sources. Note that Zn(CH_3)$_2$ was used as a 1M solution in heptanes. NMR spectra were recorded at room temperature on a Bruker DPX 300 or a Bruker Avance 300 spectrometer using the solvent as the internal lock. ^1H and $^{13}C\{^1H\}$ signals are referred to residual solvent signals, those of $^{31}P\{^1H\}$ to 85% H_3PO_4 and the ^{19}F NMR spectra to external $CFCl_3$. ^1H, ^{19}F, $^{31}P\{^1H\}$ and $^{13}C\{^1H\}$ NMR signal assignments were supported or, when stated, determined by $^1H,^1H$ COSY, $^{19}F,^1H$ HETCOR, $^{19}F,^{19}F$ gCOSY, $^{31}P,^{31}P$ COSY, $^1H,^{13}C$ HMQC, $^1H,^{13}C$ HMBC, $^{19}F,^{13}C$ HMBC, $^{19}F,^{13}C$ HMQC and $^1H,^{31}P$ HMBC experiments. The determined coupling constant values were confirmed by *gNMR* software simulations.[7] GC-MS was measured with an Agilent 6890N gas-phase chromatograph (Agilent 19091S-433 Hewlett-Packard) with an Agilent 5973 Network mass selective detector at 70 eV. High Resolution Electron Impact Mass Spectra (HR-MS, EI) were measured with an Orbitrap Exploris GC 240 at 70eV with a Trace 1310 GC and a Thermo Scientifice Trace Gold TG-5SILMS column.

1.2 Reaction of [Rh{(*E*)-CF=CHCF$_3$}(PEt_3)$_3$] (1) with Zn(CH_3)$_2$

In a Young NMR tube [Rh{(*E*)-CF=CHCF$_3$}(PEt_3)$_3$] (**1**) (25 mg, 0.044 mmol) was dissolved in C_6D_6 (0.4 mL). Then, one equivalent of Zn(CH_3)$_2$ (44 µl, 0.044 mmol) was added. The reaction was monitored by NMR spectroscopy. After 20 minutes at room temperature a mixture of [Rh(CH_3)($ZnCH_3$){(*Z*)-C(CH_3)=CHCF$_3$}(PEt_3)$_2$] (**2**), [Rh{(*E*)-C(CH_3)=CHCF$_3$}(PEt_3)$_3$] (*E*-**3**), [Rh{(*Z*)-C(CH_3)=CHCF$_3$}(PEt_3)$_3$] (*Z*-**3**) and [Rh(C≡CCF_3)(PEt_3)$_3$] (**4**) was observed in a 3:0.7:2.5:1 ratio, together with an unknown compound. After 1d, the ratio changed to 0.4:3.6:0.9:1.

Alternatively, Zn(CH_3)$_2$ was added to a toluene-d$_8$ (0.4 mL) solution of complex **1** at 243K and the solution was slowly warmed-up to 253K. After 4h, 75% of complex **2** together with

a possible isomer (9:1) and free phosphine were observed. After warming up to room temperature, the same result as above was obtained.

The preparation of [Rh(^{13}CH$_3$)(Zn^{13}CH$_3$){(*Z*)-C(^{13}CH$_3$)=CHCF$_3$}(PEt$_3$)$_2$] (**2'**) was performed following the procedure at low temperature by using previously synthesized Zn(^{13}CH$_3$)$_2$ dissolved in pentane.

Analytical data for **2**: **^{1}H NMR** (300.1 MHz, C$_6$D$_6$): δ = 6.18 (q br, $^3J_{H,F}$ = 8.5 Hz, 1H, =C*H*); 2.24 (qddt, t br in ^{1}H{^{19}F} NMR spectrum, qdd in ^{1}H{^{31}P} NMR spectrum, $^3J_{H,F}$ = 3.0, $^4J_{H,H}$ ≈ $^3J_{H,Rh}$ = 1.4, $^4J_{H,P}$ = 0.5 Hz, 3H, =C-C*H$_3$*); -0.15 (s, 3H, Zn-C*H$_3$*); -0.33 (t, $^3J_{H,P}$ = 4.6 Hz, 3H, Rh-C*H$_3$*) ppm; the resonances corresponding to the phosphine ligands are overlapped with the signals of other products. **^{19}F NMR** (282.4 MHz, C$_6$D$_6$): δ = –57.1 (m, td in ^{19}F{^{1}H} NMR spectrum, $^5J_{F,P}$ = 4, $^4J_{F,Rh}$ = 3 Hz, C*F*$_3$) ppm. **^{31}P{^{1}H} NMR** (121.5 MHz, C$_6$D$_6$): δ = 16.9 (dq, $^1J_{P,Rh}$ = 129.0, $^5J_{P,F}$ = 4.4 Hz, PEt$_3$) ppm. **^{1}H,^{13}C HMQC** and **^{1}H,^{13}C HMBC** (300.1/75.5 MHz, toluene-d^8, 253K): δ = 193 (=*C*-CH$_3$); 127 (m, $^1J_{C,F}$ = 294 Hz, CF$_3$); 115 (=CH); 35 (=C-*C*H$_3$); -9 (Zn-CH$_3$); -28 (Rh-CH$_3$) ppm.

Selected NMR data for **2'**: **^{1}H NMR** (300.1 MHz, toluene-d^8, 253K): δ = -0.21 (d, $^1J_{H,C}$ = 123.2 Hz, Zn-C*H$_3$*); -0.42 (dt, $^1J_{H,C}$ = 121.1, $^3J_{H,P}$ = 4.6 Hz, 3H, Rh-C*H$_3$*) ppm;. **^{31}P{^{1}H} NMR** (121.5 MHz, toluene-d^8, 253K): δ = 16.9 (dpd, $^1J_{P,Rh}$ = 129.0, $^5J_{P,F}$ = $^2J_{P,C}$ = 4.6, $^3J_{P,C}$ = 1.5 Hz, PEt$_3$) ppm. **^{13}C{^{1}H} NMR** (75.5 MHz, toluene-d^8, 253K): δ = 34.4 (dt, $^2J_{C,Rh}$ = 3.3, $^3J_{C,P}$ = 1.7 Hz, =C-*C*H$_3$); -9.2 (t br, $^2J_{C,Rh}$ = $^3J_{C,C}$ = 5.2 Hz, Zn-CH$_3$); -27.5 (dq, $^2J_{C,Rh}$ = 9.3, $^3J_{C,P}$ = $^3J_{C,C}$ = 5.2 Hz, Rh-CH$_3$) ppm.

Analytical data for *E*-**3**: **^{1}H NMR** (300.1 MHz, C$_6$D$_6$): δ = 5.69 (pent br, dm in ^{1}H{^{19}F} NMR spectrum, q pseudo t in ^{1}H{^{31}P} NMR spectrum, $^3J_{H,F}$ = 9.6, $^4J_{H,Ptrans}$ = 7.4, $^4J_{H,H}$ ≈ $^3J_{H,Rh}$ = 1.3, $^4J_{H,Pcis}$ = 1.9 Hz, 1H, =C*H*); 2.65 (pm, dd in ^{1}H{^{19}F} NMR spectrum, qd in ^{1}H{^{31}P} NMR spectrum, $^4J_{H,Ptrans}$ = 3.7, $^3J_{H,F}$ = 2.8, $^4J_{H,H}$ = 1.3, Hz, 3H, =C-C*H$_3$*) ppm; the resonances corresponding to the phosphine ligands are overlapped with the signals of other products. **^{19}F NMR** (282.4 MHz, C$_6$D$_6$): δ = –55.1 (dtq, $^3J_{F,H}$ = 10, $^5J_{F,P}$ = 5, $^5J_{F,H}$ = 3 Hz, C*F*$_3$) ppm. **^{31}P{^{1}H} NMR** (121.5 MHz, C$_6$D$_6$): δ = 19.4 (dt, $^1J_{P,Rh}$ = 114.4, $^3J_{P,P}$ = 35.6 Hz, P$_{trans}$); 12.6 (ddq, $^1J_{P,Rh}$ = 165.1, $^2J_{P,P}$ = 35.6, $^3J_{P,F}$ = 5.5 Hz, P$_{cis}$) ppm. **^{1}H,^{13}C HMQC** and **^{1}H,^{13}C HMBC** (300.1/75.5 MHz, C$_6$D$_6$): δ = 199 (=*C*-CH$_3$); 123 (m, $^1J_{C,F}$ = 270 Hz, CF$_3$); 117 (=CH); 21 (=C-*C*H$_3$) ppm.

Selected NMR data for *E*-**3'**: **^{1}H NMR** (300.1 MHz, toluene-d^{8}): δ = 2.65 (d br, $^{1}J_{H,C}$ = 123.0 Hz, =C-C*H$_3$*) ppm **^{31}P{^{1}H} NMR** (121.5 MHz, toluene-d^{8}): δ = 19.4 (dt, $^{1}J_{P,Rh}$ = 114.4, $^{3}J_{P,P}$ = 35.6 Hz, P_{trans}); 12.6 (ddqd, $^{1}J_{P,Rh}$ = 130.1, $^{2}J_{P,P}$ = 35.6, $^{3}J_{P,F}$ = 5.5, $^{3}J_{P,C}$ = 1.8 Hz, P_{cis}) ppm. **^{13}C{^{1}H} NMR** (75.5 MHz, toluene-d^{8}): δ = 20.8 (q, $^{2}J_{C,Rh}$ = $^{3}J_{C,P}$ = 2.0 Hz, =C-*C*H$_3$) ppm.

Analytical data for *Z*-**3**: **^{1}H NMR** (300.1 MHz, C_6D_6): δ = 6.32 (pent br, dm in ^{1}H{^{19}F} NMR spectrum, qm in ^{1}H{^{31}P} NMR spectrum, $^{4}J_{H,Ptrans}$ = 10.6, $^{3}J_{H,F}$ = 8.9 Hz, 1H, =C*H*); 2.39 (pm, dd in ^{1}H{^{19}F} NMR spectrum, qd in ^{1}H{^{31}P} NMR spectrum, $^{4}J_{H,Ptrans}$ = 2.9, $^{3}J_{H,F}$ = 2.7, $^{4}J_{H,H}$ = 1.1, Hz, 3H, =C-C*H$_3$*) ppm; the resonances corresponding to the phosphine ligands are overlapped with the signals of other products. **^{19}F NMR** (282.4 MHz, C_6D_6): δ = –56.0 (dtqd, $^{3}J_{F,H}$ = 9, $^{5}J_{F,P}$ = 5, $^{5}J_{F,H}$ = 3, $J_{F,P/Rh}$ = 2 Hz, C*F*$_3$) ppm. **^{31}P{^{1}H} NMR** (121.5 MHz, C_6D_6): δ = 14.2 (dt br, $^{1}J_{P,Rh}$ = 112.3, $^{3}J_{P,P}$ = 35.6 Hz, P_{trans}); 6.8 (ddq, $^{1}J_{P,Rh}$ = 156.5, $^{2}J_{P,P}$ = 36.7, $^{3}J_{P,F}$ = 5.3 Hz, P_{cis}) ppm. **^{1}H,^{13}C HMQC** and **^{1}H,^{13}C HMBC** (300.1/75.5 MHz, C_6D_6): δ = 195 (=*C*-CH$_3$); 127 (m, $^{1}J_{C,F}$ = 270 Hz, CF$_3$); 119 (=CH); 32 (=C-*C*H$_3$) ppm.

Selected NMR data for *Z*-**3'**: **^{1}H NMR** (300.1 MHz, toluene-d^{8}): δ = 2.40 (d br, $^{1}J_{H,C}$ = 123.9 Hz, =C-C*H$_3$*) ppm **^{31}P{^{1}H} NMR** (121.5 MHz, toluene-d^{8}): δ = 14.2 (dt br, $^{1}J_{P,Rh}$ = 112.3, $^{3}J_{P,P}$ = 35.6 Hz, 1P, P_{trans}); 6.8 (ddqd, $^{1}J_{P,Rh}$ = 156.5, $^{2}J_{P,P}$ = 36.7, $^{3}J_{P,F}$ = 5.3, $^{3}J_{P,C}$ = 1.8 Hz, P_{cis}) ppm. **^{13}C{^{1}H} NMR** (75.5 MHz, toluene-d^{8}): δ = 31.7 (t br, $^{3}J_{C,P}$ = 1.9 Hz, =C-*C*H$_3$) ppm.

1.3 General procedure for the catalytic Negishi coupling at fluoroolefins

In a Young NMR tube [Rh(CH$_3$)(PEt$_3$)$_3$] (**7**) (8 mg, 0.018 mmol) was dissolved in C_6D_6 or THF-d$_8$ (0.3 mL, 0.4 ml for the reaction with $ZnPh_2$) and the corresponding Zn reagent was added (0.18 mmol). Note that $Zn(CH_3)_2$ and $ZnEt_2$ have been added as 1M solutions in heptane and hexane, respectively. The reaction mixture was frozen to 77 K, the NMR tube was degassed *in vacuo*, and pressurized with the fluoroolefin to 0.25 bar (0.30 mmol). The yields were determined by ^{19}F NMR data using trifluorotoluene both as internal and external standard.

Table S1. Negishi cross-coupling at fluorinated olefins

Olefin	Solvent	Time	Yield (%)	Products
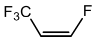 **5a**	C_6D_6	1h	99	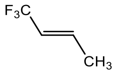 **6a**
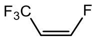 **5a**	THF-d_8	7d	99	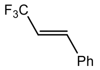 **8a**
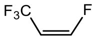 **5a**	THF-d_8	1d	60	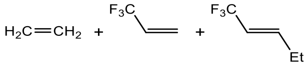 **9a**<br>5 : 2.3 : 1
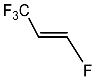 **5b**	C_6D_6	6d	95	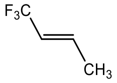 **6a**
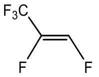 **5c**	THF-d_8	1d	25[b]	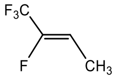 **6c**
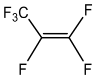 **5d**	THF-d_8	1d	10	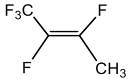 ***E*-6d** 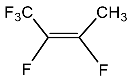 ***Z*-6d**<br>0.9 : 1
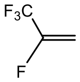 **5e**	THF-d_8	6d	11	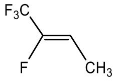 **6c**
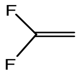 **5f**	C_6D_6	4d[a]	Traces	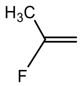 **6f**
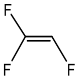 **5g**	THF-d_8	1d	17	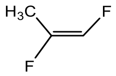 **6g**

[a] At 60ºC. [b] Main product

Compound **6a**: **^{1}H NMR** (300.1 MHz, THF-d_8): δ = 6.40 (dqq, $^{3}J_{H,H}$ = 15.6, $^{3}J_{H,H}$ = 6.6, $^{4}J_{H,F}$ = 2.3 Hz, 1H, =C*H*CH$_3$); 5.72 (dqq, $^{3}J_{H,H}$ = 15.6, $^{3}J_{H,F}$ = 6.4, $^{4}J_{H,H}$ = 1.7 Hz, 1H, =C*H*CF$_3$); 1.80 (dqd, $^{3}J_{H,H}$ = 6.7, $^{5}J_{H,F}$ = 2.7, $^{4}J_{H,H}$ = 1.9, Hz, 3H, C*H$_3$*) ppm. **^{19}F NMR** (282.4 MHz, THF-d_8): δ = –65.1 (d pseudo p, $^{3}J_{F,H}$ = 6, $^{5}J_{F,H} \approx {}^{4}J_{F,H}$ = 2 Hz, C*F*$_3$) ppm. **^{13}C{^{1}H} NMR** (75.5 MHz, C_6D_6): δ = 136.2 (q, $^{3}J_{C,F}$ = 6.8 Hz, =*C*HCH$_3$); 123.9 (q, $^{1}J_{C,F}$ = 268.6 Hz, CF$_3$); 120.4 (q, $^{2}J_{C,F}$ = 33.2 Hz,=*C*HCF$_3$); 17.1 (s, *C*H$_3$) ppm. **GC/MS**: 110 [M]$^{+}$.

HR-MS (EI): $C_4H_5F_3$ $[M]^+$, calculated *m/z* 110.0343 (100%), 111.0343 (4.40%); found *m/z* 110.0337 (100%), 111.0371 (4.07%).

Compound **6c**: **^{1}H NMR** (300.1 MHz, THF-d_8): δ = 5.72 (dq, $^3J_{H,F}$ = 33.7, $^3J_{H,H}$ = 6.7 Hz, 1H, =C*H*); 1.72 (m, 3H, C*H₃*) ppm. **^{19}F NMR** (282.4 MHz, THF-d_8): δ = –75.4 (dq, $^3J_{F,F}$ = 12, $^5J_{F,H}$ = 2 Hz, 3F, CF_3); -141.3 (ddq, $^3J_{F,H}$ = 34, $^3J_{F,F}$ = 12, $^4J_{F,H}$ = 3 Hz, 1F, =C*F*) ppm. **GC/MS**: 128 $[M]^+$, 119 $[M\text{-}F]^+$.

Compound *E*-**6d**: **^{1}H NMR** (300.1 MHz, THF-d_8): δ = 1.91-1.85 (m, CH_3) ppm. **^{19}F NMR** (282.4 MHz, THF-d_8): δ = –68.9 (dd br, $^4J_{F,F}$ = 21, $^3J_{F,F}$ = 11 Hz, 3F CF_3); -132.5 (dqq, $^3J_{F,F}$ = 133, $^4J_{F,F}$ = 21, $^3J_{F,H}$ = 4 Hz, 1F, =C*F*CH_3); -176.4 (dqq, $^3J_{F,F}$ = 133, $^3J_{F,F}$ = 11, $^4J_{F,H}$ = 6 Hz, 1F, =C*F*CF_3) ppm.

Compound *Z*-**6d**: **^{1}H NMR** (300.1 MHz, THF-d_8): δ = 2.18 (ddq, $^3J_{H,F}$ = 19.7, $^4J_{H,F}$ = 7.3, $^5J_{H,F}$ = 2.3 Hz, 3H, CH_3) ppm. **^{19}F NMR** (282.4 MHz, THF-d_8): δ = –67.8 (ddq, $^3J_{F,F}$ = 12, $^4J_{F,F}$ = 9, $^5J_{F,H}$ = 2 Hz, 3F, CF_3); -112.8 (qqd, $^3J_{F,H}$ = 19, $^4J_{F,F}$ = 9, $^3J_{F,F}$ = 5 Hz, 1F, =C*F*CH_3); -161.7 (qqd, $^3J_{F,F}$ = 12, $^4J_{F,H}$ = 7, $^3J_{F,F}$ = 5 Hz, 1F, =C*F*CF_3) ppm.

Compound **6f**: [8] **^{1}H NMR** (300.1 MHz, C_6D_6): δ = 1.80 (dm, $^3J_{H,F}$ = 16.3 Hz, 3H, CH_3); 4.95 (dm, $^3J_{H,F}$ = 33.0 Hz, CHtrans to F); 5.20 (m, CH*cis* to F) ppm. **^{19}F NMR** (282.4 MHz, C_6D_6): δ = –88.2 (dq, $^3J_{F,H}$ = 32, $^3J_{F,H}$ = 17 Hz, 1F, C*F*) ppm.

Compound *E*-**6g**: **^{1}H NMR** (300.1 MHz, THF-d_8): δ = 7.19 (ddq, $^2J_{H,F}$ = 77.0, $^3J_{H,F}$ = 3.2, $^4J_{H,H}$ = 1.3 Hz, 1H, =C*H*); 1.96 (ddd, $^3J_{H,F}$ = 17.7, $^4J_{H,F}$ = 5.5, $^4J_{H,H}$ = 1.3 Hz, 3H, CH_3) ppm. **^{19}F NMR** (282.4 MHz, THF-d_8): δ -153.9 (dqd, $^3J_{F,F}$ = 128, $^3J_{F,H}$ = 17, $^3J_{F,H}$ = 4 Hz, 1F,=C*F*CH_3); -184.4 (dqd, $^3J_{F,F}$ = 128, $^3J_{F,H}$ = 77, $^4J_{F,H}$ = 6 Hz, 1F,=C*F*H) ppm. **GC/MS**: 78 $[M]^+$.

Compound **8a**[9] : **^{1}H NMR** (300.1 MHz, THF-d_8): δ = 7.30-6.60 (m, 5H, Ph); 7.29 (dq, $^4J_{H,H}$ = 16.1, $^3J_{H,F}$ = 2.3 Hz, 1H, =C*H*Ph); 6.53 (dq, $^3J_{H,H}$ = 16.1, $^3J_{H,F}$ = 6.4 Hz, 1H, =C*H*CF_3) ppm. **^{19}F NMR** (282.4 MHz, THF-d_8): δ = –64 (m, dd in C_6D_6, $^3J_{F,H}$ = 6, $^5J_{F,H} \approx {}^4J_{F,H}$ = 2 Hz, CF_3) ppm. **GC/MS**: 172 $[M]^+$. **HR-MS (EI)**: $C_9H_7F_3$ $[M]^+$, calculated *m/z* 172.0499 (100%), 173.0499 (9.84%); found *m/z* 172.0494 (100%), 173.0528 (9.32%).

Compound **9a**: **^{1}H NMR** (300.1 MHz, THF-d_8): δ = 6.72 (d br, $^{3}J_{H,H}$ = 15.1 Hz, 1H, =C*H*Et); 6.42 (dq, $^{3}J_{H,H}$ = 15.3, $^{3}J_{H,F}$ = 6.7 Hz, 1H, =C*H*CF_3) ppm. The ethyl group signals are overlapped with other signals. **^{19}F NMR** (282.4 MHz, THF-d_8): δ = –67.4 (d br, partially overlapped with 3,3,3-trifluoropropene, $^{3}J_{F,H}$ = 6 Hz, CF_3) ppm. **GC/MS**: 124 $[M]^+$.

1.4 Optimization attempts

The catalytic process was studied under different conditions (Table S2) in order to improve the conversion. *Z*-1,2,3,3,3-pentafluoropropene (**5c**) was chosen for these optimization attempts due to the low but appreciable conversion observed (25%) at room temperature in THF-d_8 after 1 day. All reactions were done using 10% mol catalyst and yields for the formation of **6c** were calculated based on NMR spectroscopy. Apart from this, **5c** was also used for catalyst and methyl source's screening as described in the manuscript for the olefin **5a**, but no improvement of any catalytic activity was found.

Table S2. Negishi cross-coupling optimization attempts

Solvent	Time	Temperature	Yield (%)
THF-d_8	1d	25	25
THF-d_8	7d	25	28
THF-d_8	1d	50	15
THF-d_8[a]	1d	25	27
C_6D_6	1d	25	17
C_6D_6	7d	25	20
C_6D_6	1d	50	13
CD_2Cl_2	1d	25	traces

[a] Addition of 2 equivalents PEt_3, lower selectivity in ^{19}F NMR spectrum.

1.5 NMR Spectra

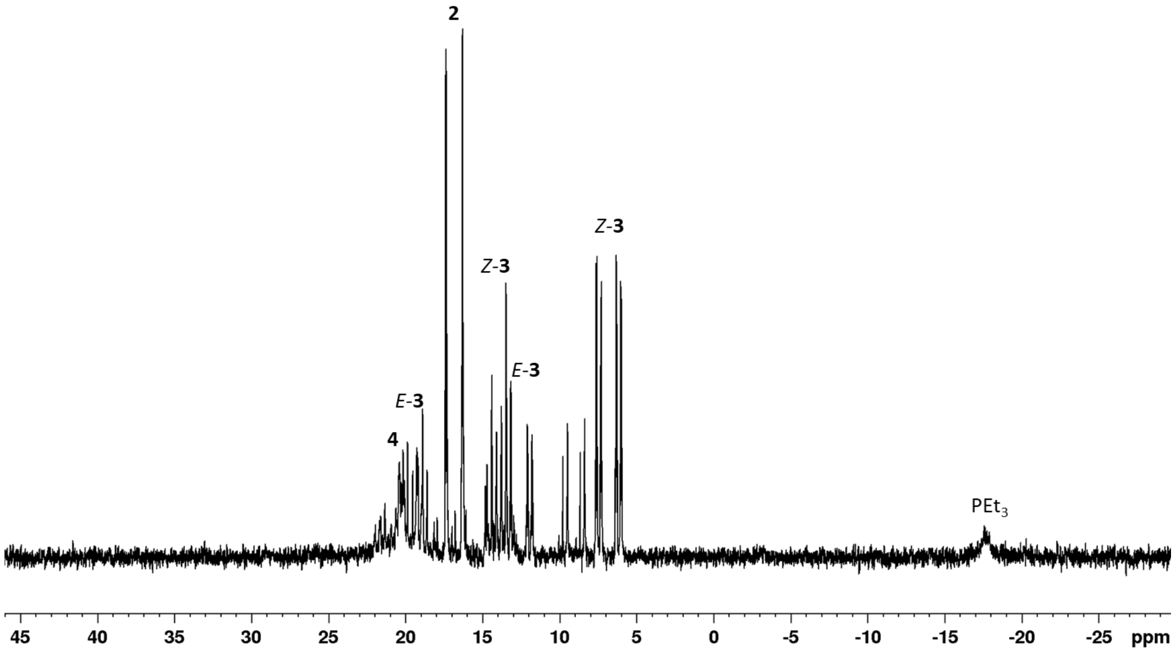

S1. $^{31}P\{^{1}H\}$ NMR spectrum of the reaction of $Zn(CH_3)_2$ with $[Rh(\textit{E}\text{-}CF{=}CHCF_3)(PEt_3)_3]$ (**1**) after 1h.

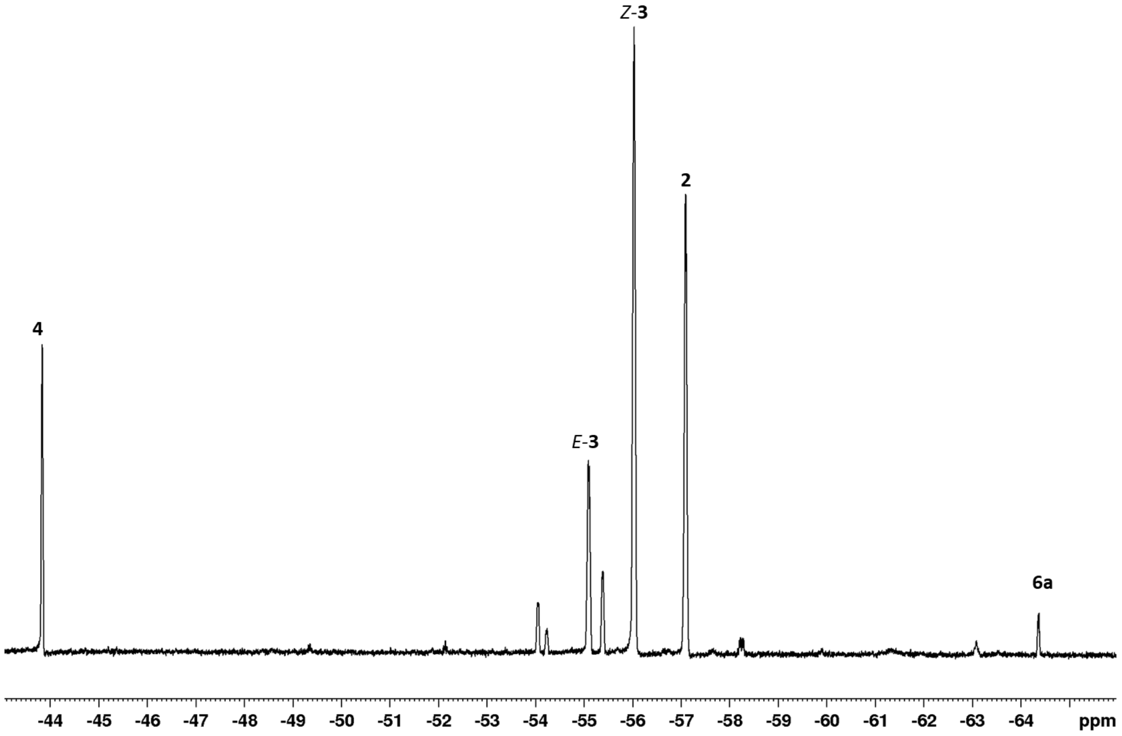

S2. ^{19}F NMR spectrum of the reaction of $Zn(CH_3)_2$ with $[Rh(\textit{E}\text{-}CF{=}CHCF_3)(PEt_3)_3]$ (**1**) after 1h.

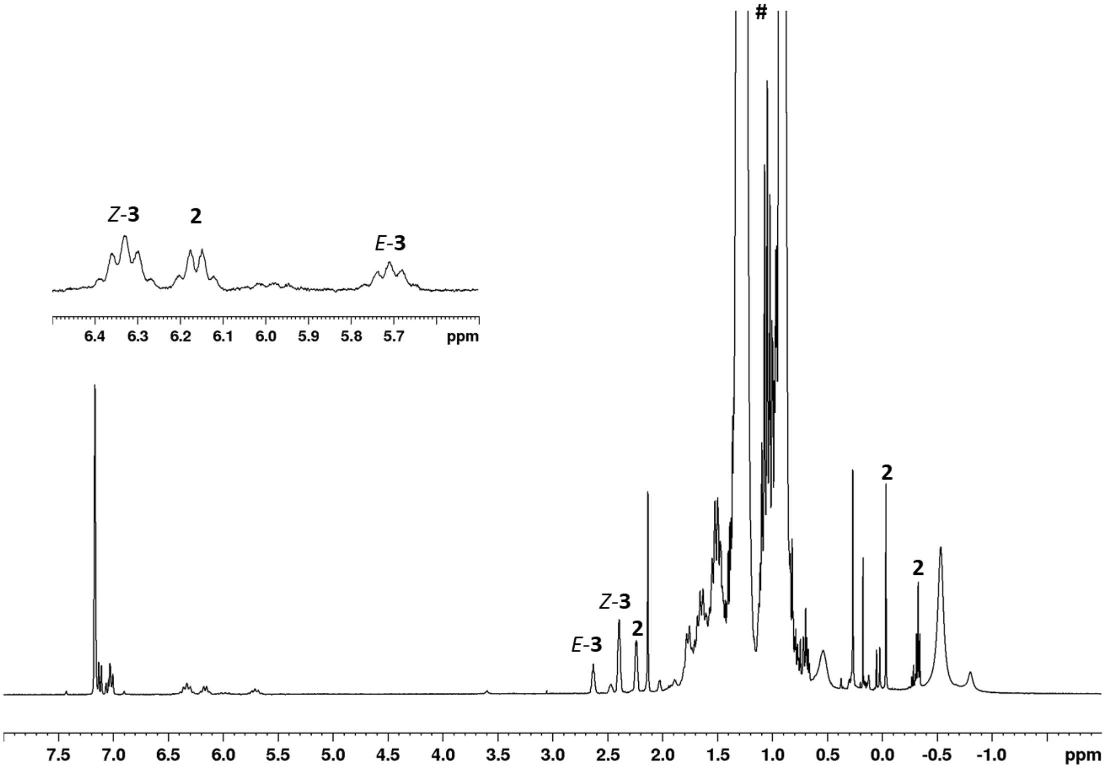

S3. ^{1}H NMR spectrum of the reaction of $Zn(CH_3)_2$ (solution in heptanes) with [Rh(*E*-CF=CHCF$_3$)(PEt$_3$)$_3$] (**1**) after 1h. # = PEt$_3$ ligands as well as the heptanes.

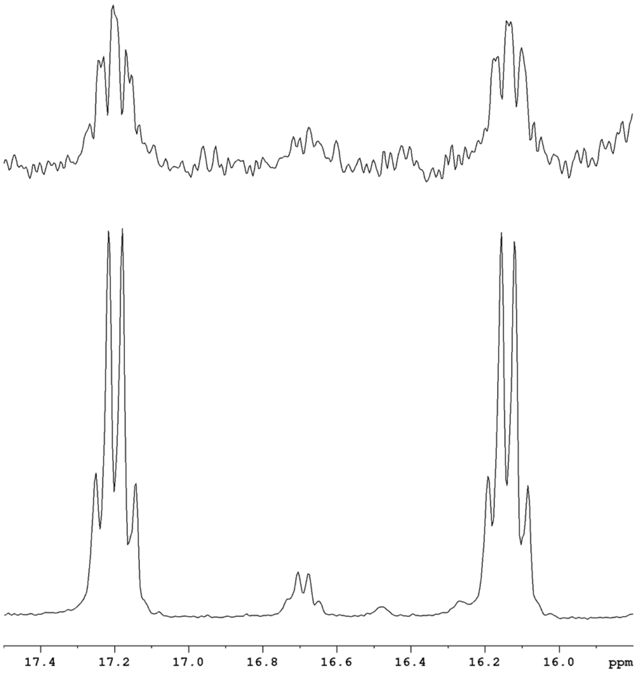

S4. Section of the $^{31}P\{^1H\}$ NMR spectrum of the reaction of $Zn(CH_3)_2$ (bottom) or $Zn(^{13}CH_3)_2$ (up) with **1** showing the formation of **2** or **2'**, respectively. The unknown peak in the middle, belongs to the probable isomer of **2** observed during the reaction.

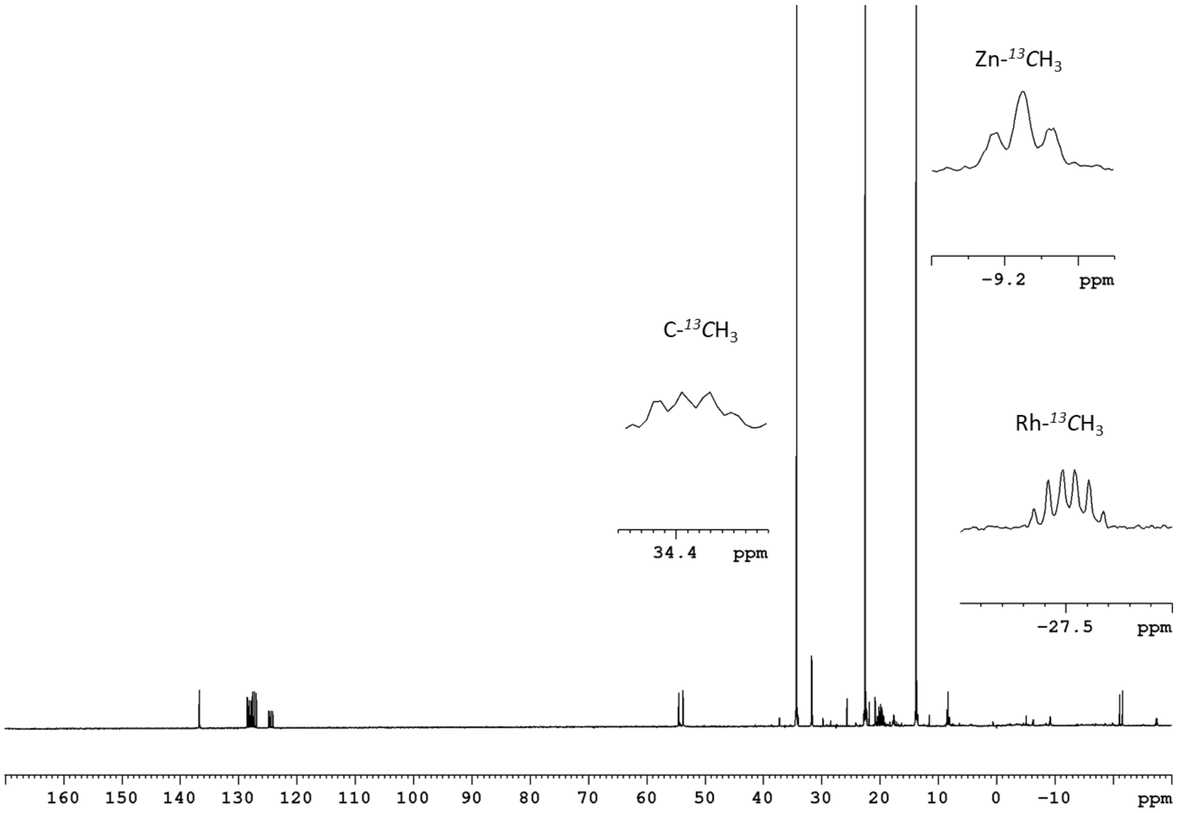

S5. $^{13}C\{^{1}H\}$ NMR spectrum of the mixture of products derived from the reaction of $Zn(^{13}CH_3)_2$ with **1**, highlighting the formation of **2’**.

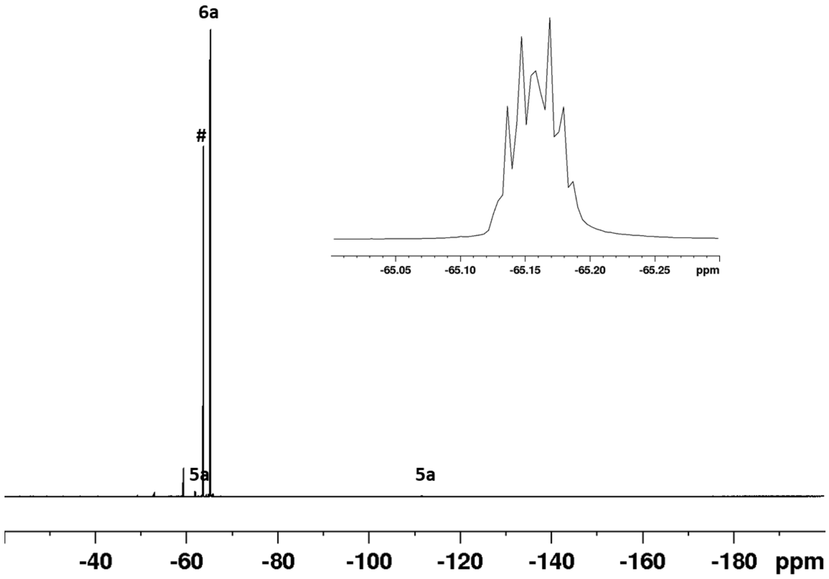

S6. ^{19}F NMR spectrum of the catalytic conversion of **5a** into **6a** in THF-d_8. **# =** trifluorotoluene

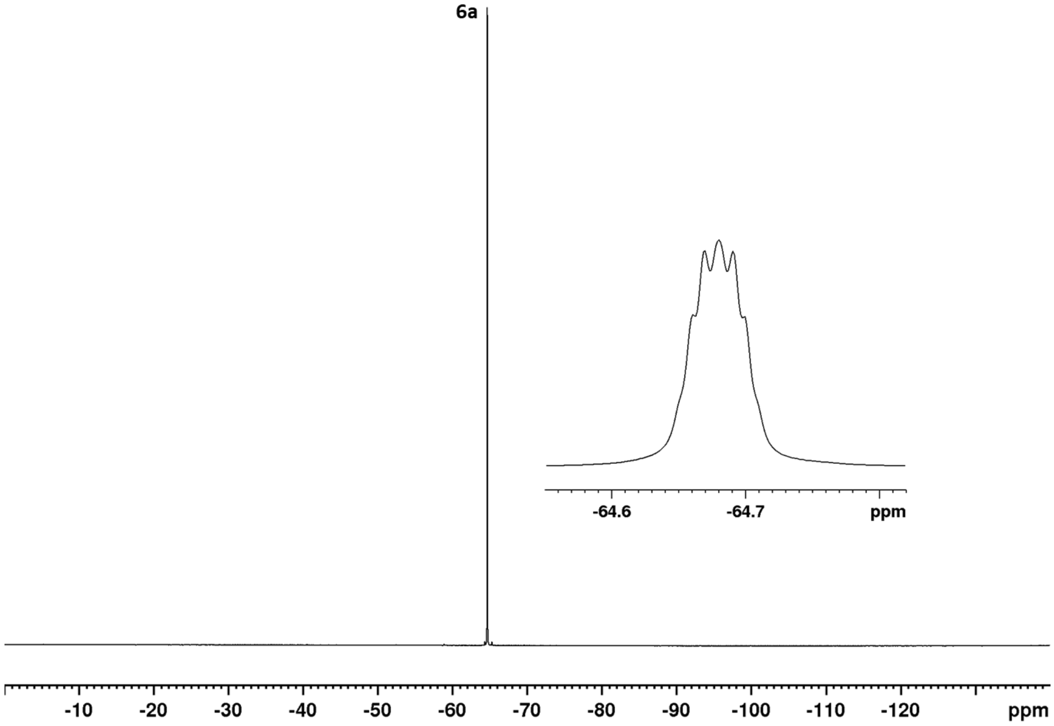

S6b. ^{19}F NMR spectrum of a clean heptane solution of **6a** in C_6D_6.

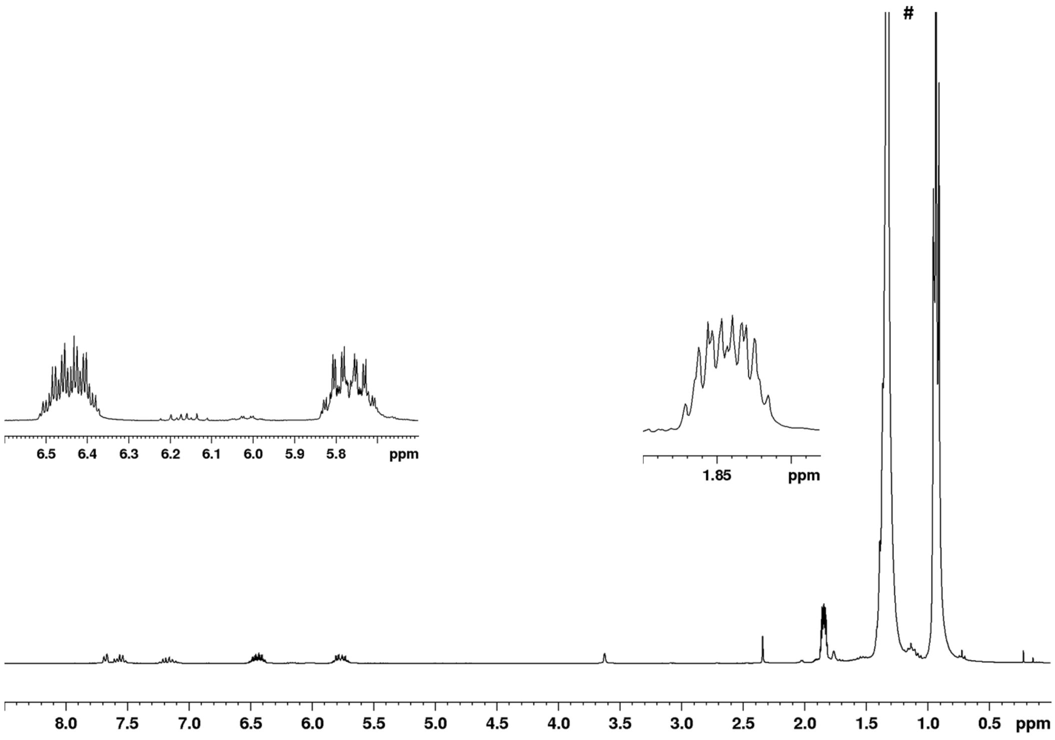

S7. ^{1}H NMR spectrum of the catalytic conversion of **5a** into **6a** in THF-d_8. **# =** heptanes.

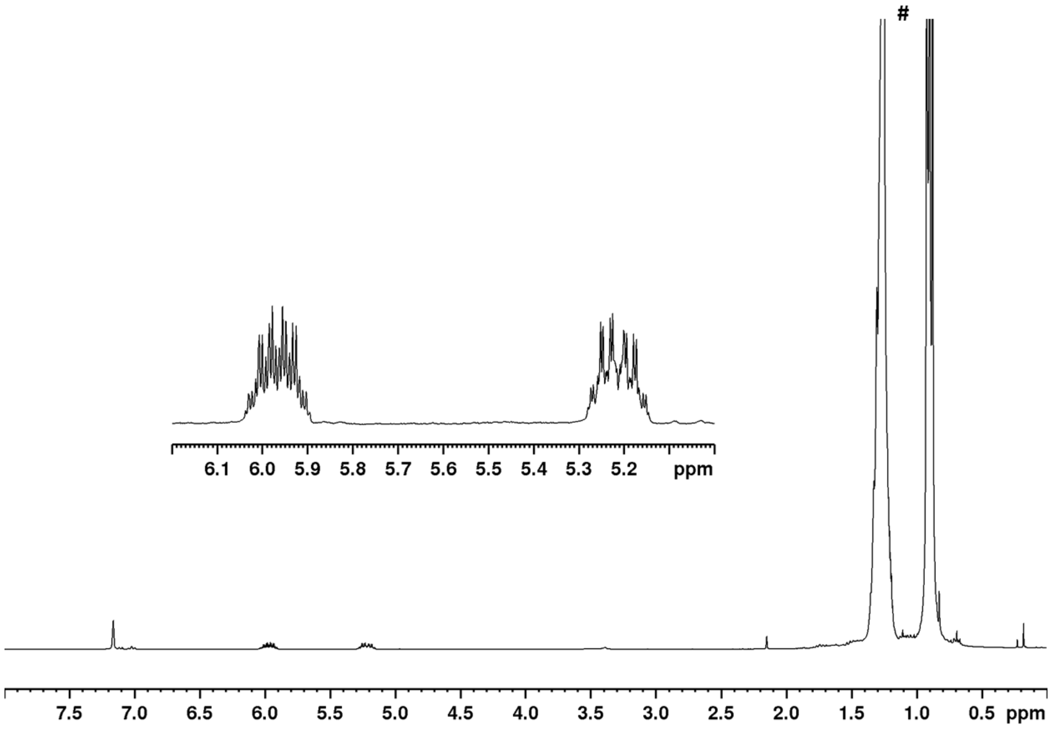

S7b. ^{1}H NMR spectrum of a clean heptane solution of **6a** in C_6D_6 (methyl signal overlapped with heptanes).

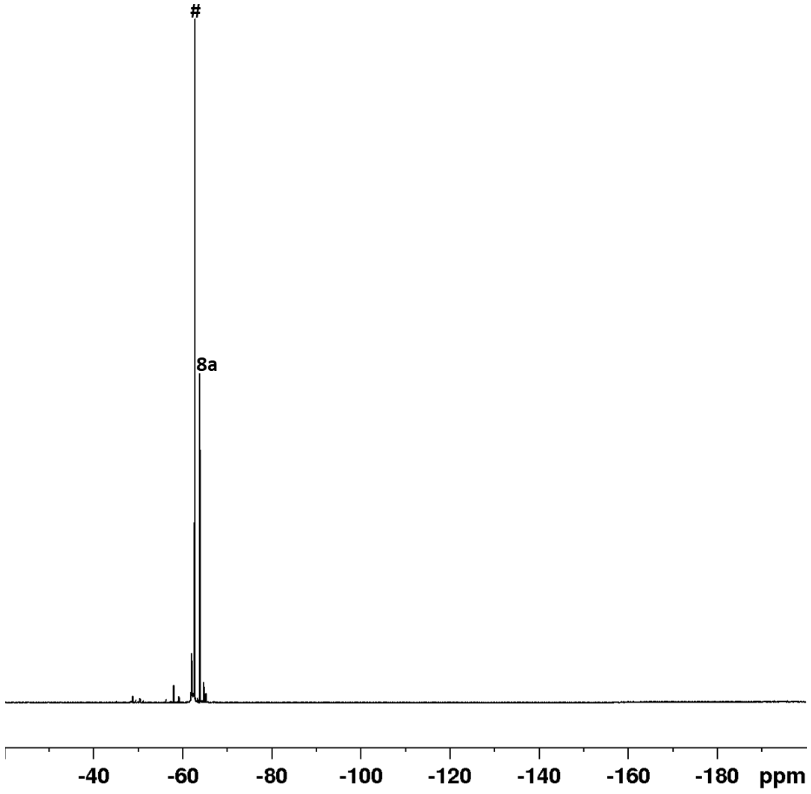

S8. ^{19}F NMR spectrum of the catalytic conversion of **5a** into **8a** in THF-d_8. # = trifluorotoluene

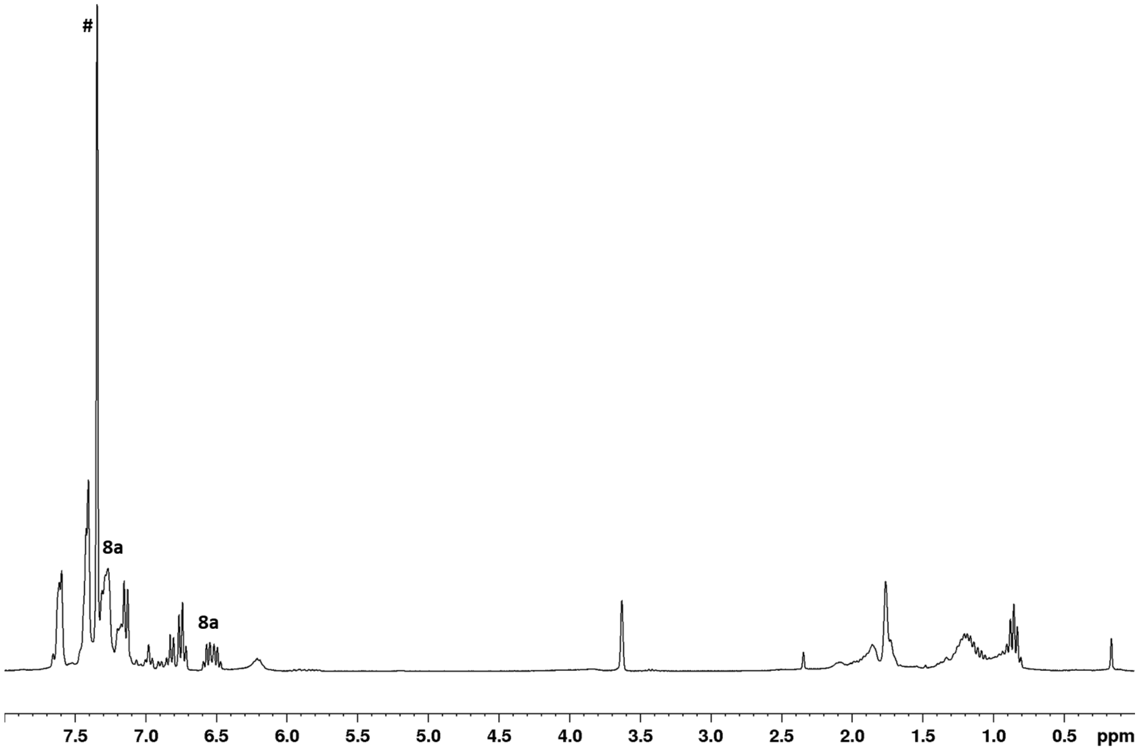

S9. ^{1}H NMR spectrum of the catalytic conversion of **5a** into **8a** in THF-d_8. **# =** trifluorotoluene

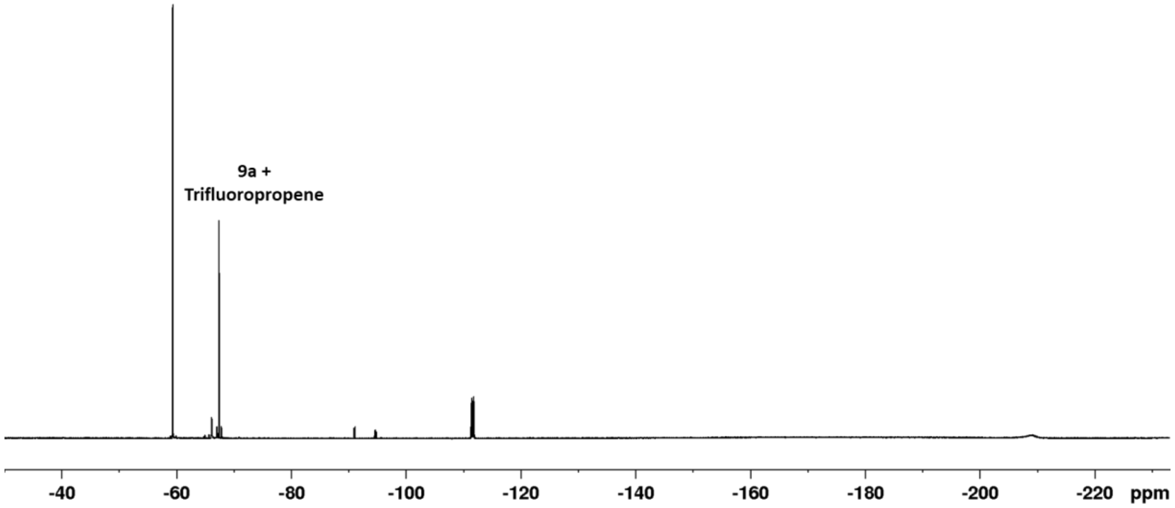

S10. ^{19}F NMR spectrum of the catalytic conversion of **5a** into **9a** in THF-d_8.

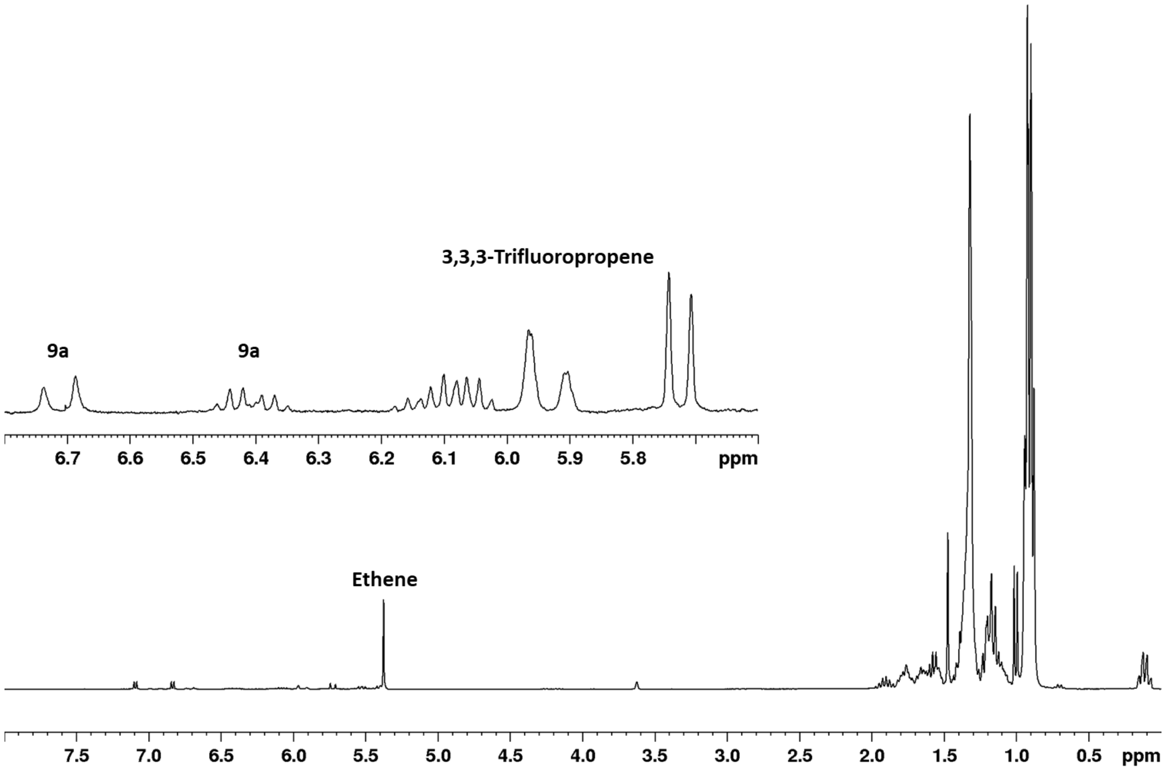

S11. ^{1}H NMR spectrum of the catalytic conversion of **5a** into **9a**, ethene and 3,3,3-trifluoropropene in THF-d_8.

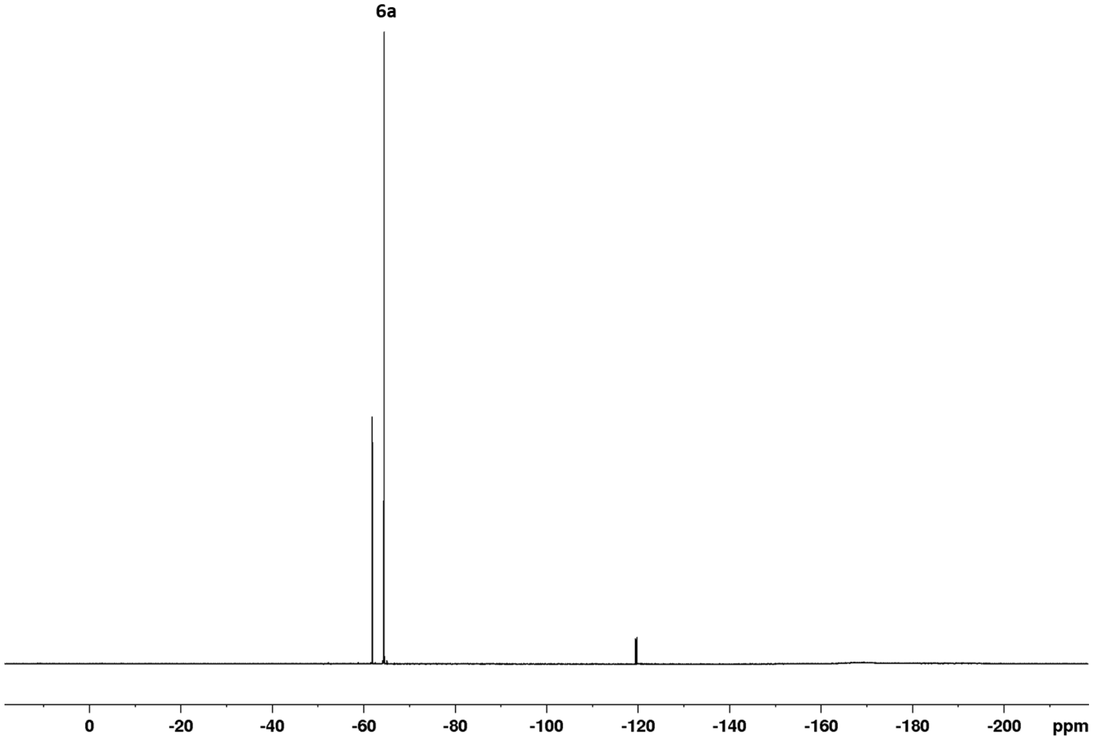

S12. ^{19}F NMR spectrum of the catalytic conversion of **6b** into **6a** in C_6D_6.

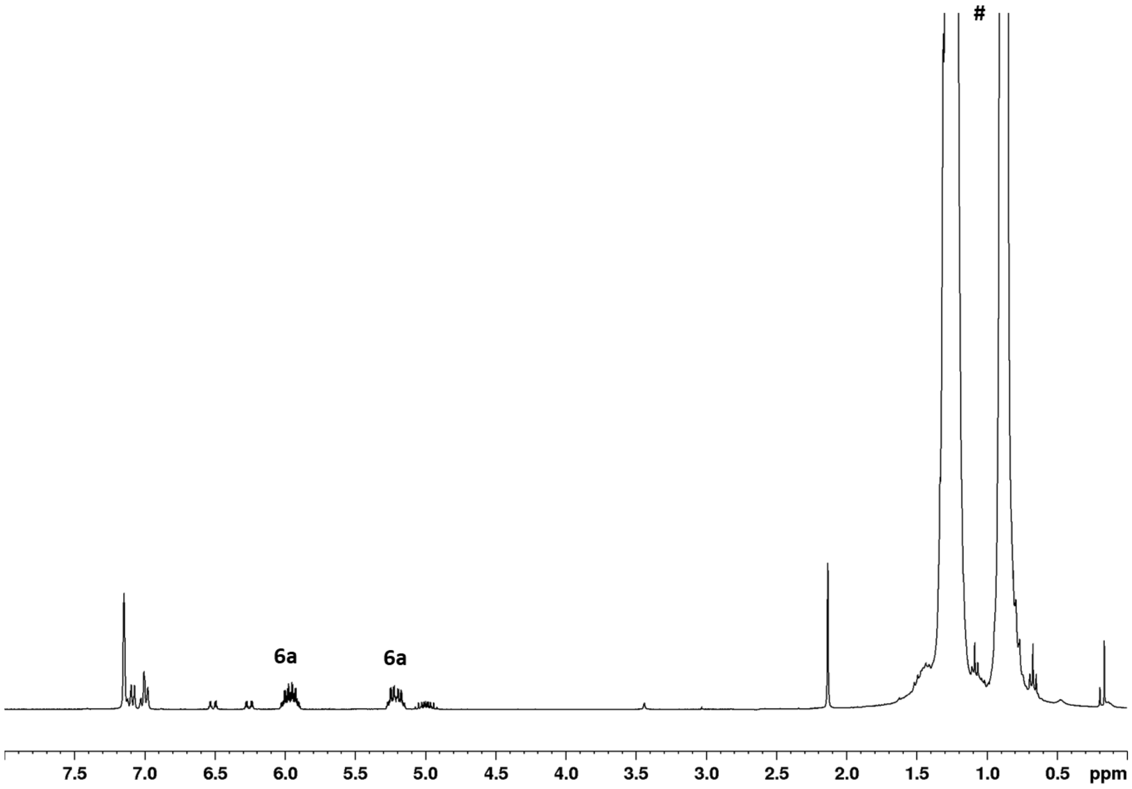

S13. ^{1}H NMR spectrum of the catalytic conversion of **6b** into **6a** in C_6D_6. **# =** heptanes

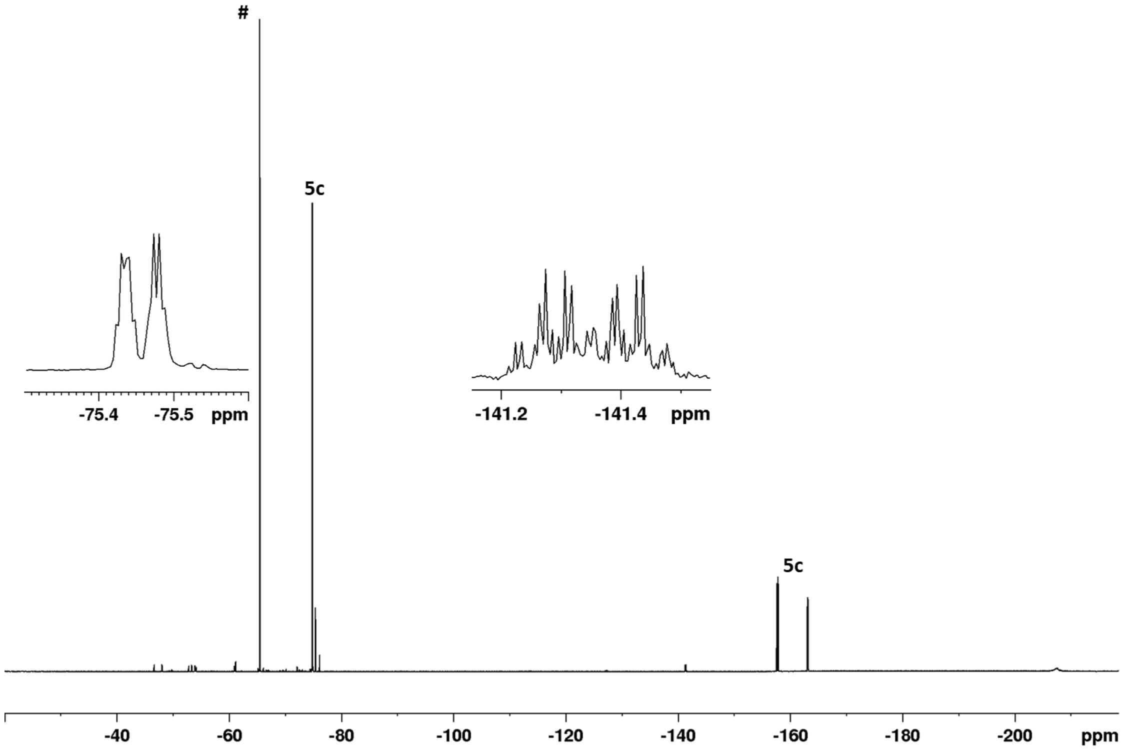

S14. ^{19}F NMR spectrum of the catalytic conversion of **5c** into **6c** in THF-d_8. **# =** trifluorotoluene

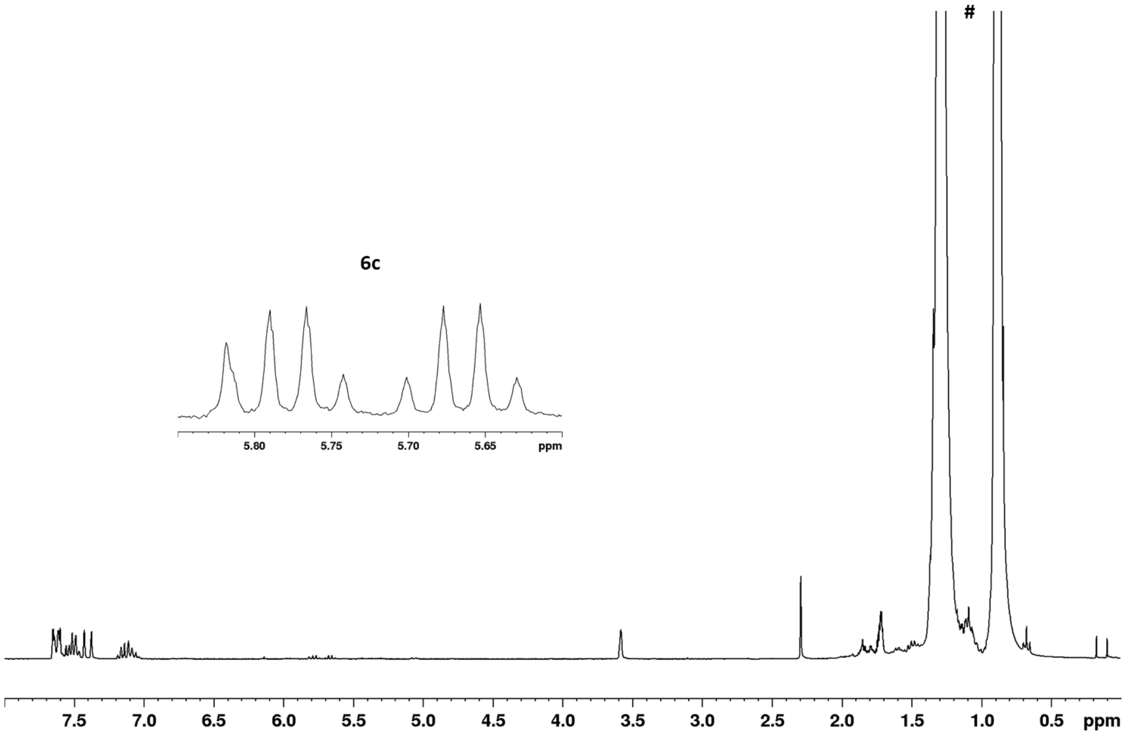

S15. ^{1}H NMR spectrum of the catalytic conversion of **5c** into **6c** in THF-d_8.

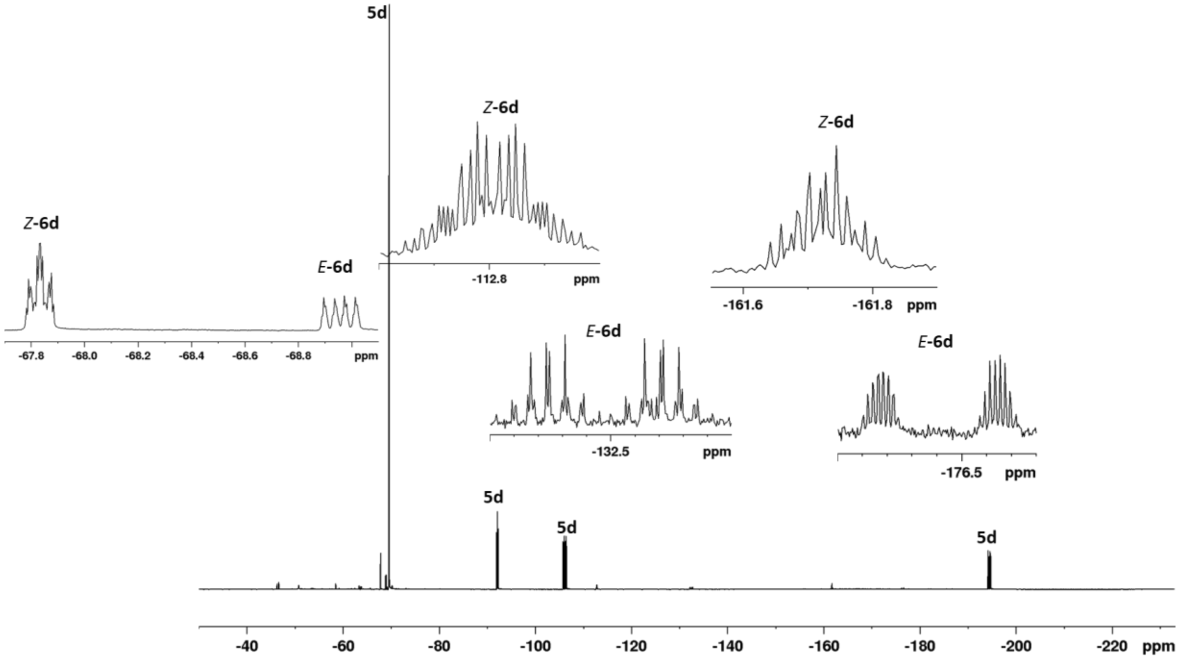

S16. ^{19}F NMR spectrum of the catalytic conversion of **5d** into **6d** in THF-d_8. **# =** trifluorotoluene

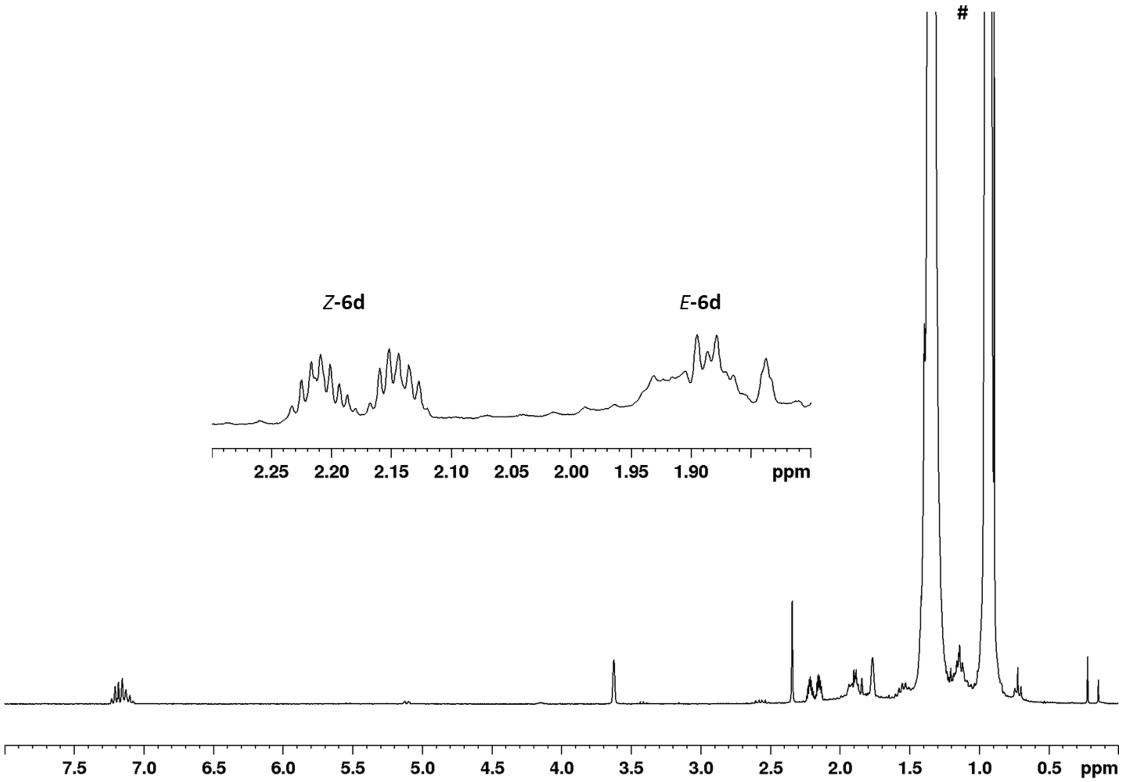

S17. ^{1}H NMR spectrum of the catalytic conversion of **5d** into **6d** in THF-d_8. # = heptanes

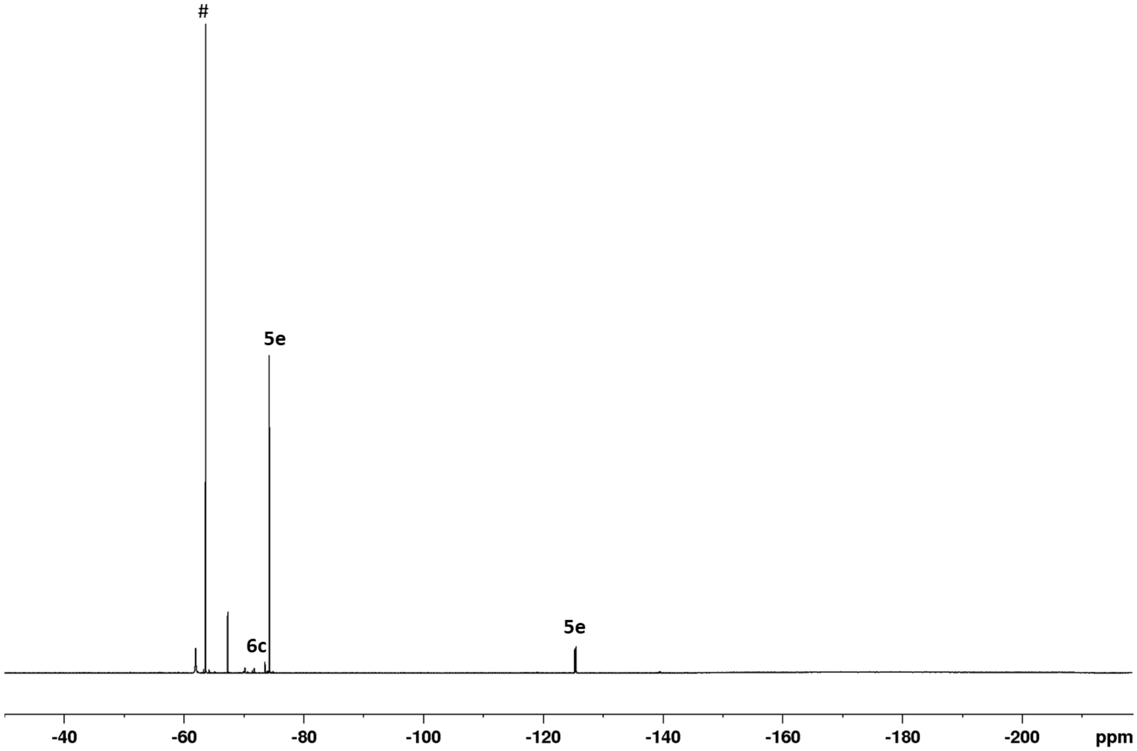

S18. ^{19}F NMR spectrum of the catalytic conversion of **5e** into **6c** and unknown compounds in THF-d_8. # = trifluorotoluene

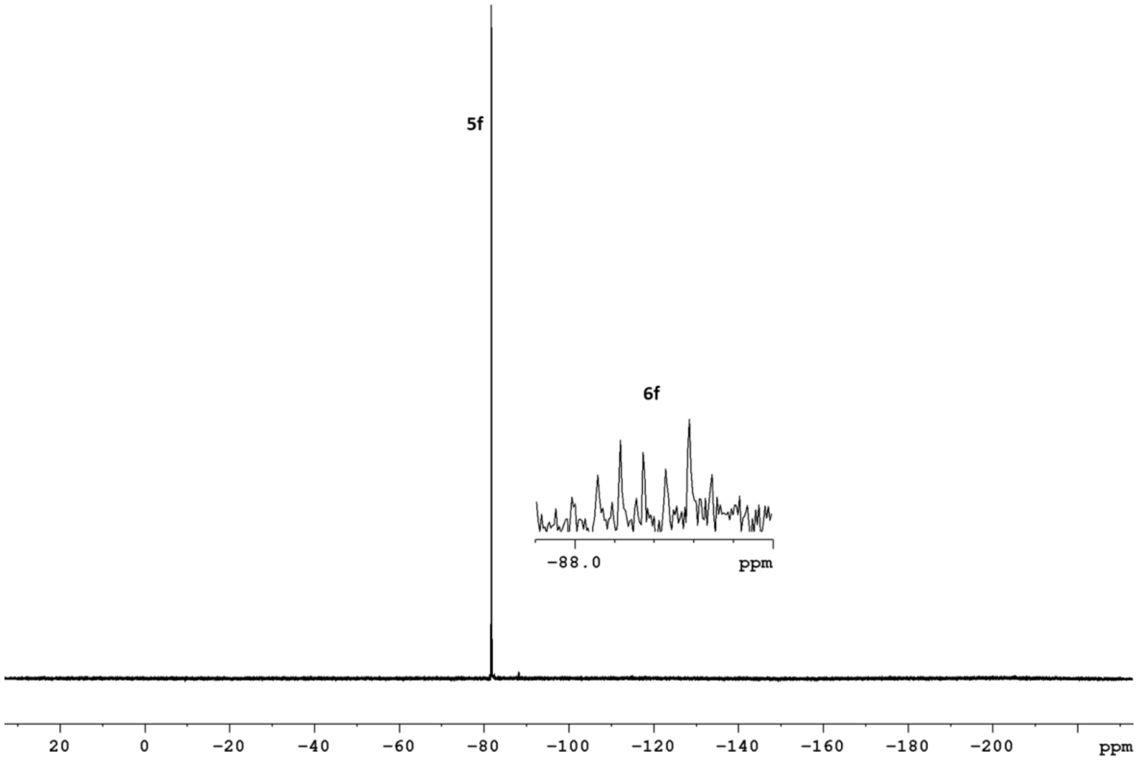

S19. ^{19}F NMR spectrum of the catalytic conversion of **5f** into **6f** in C_6D_6.

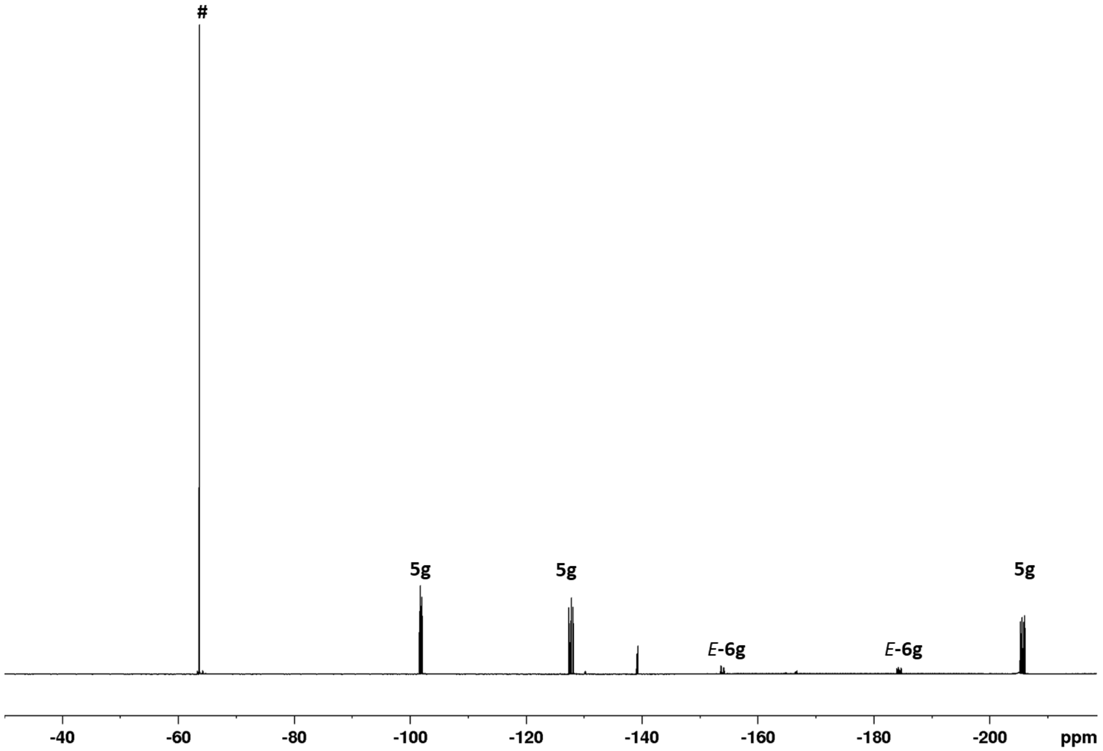

S20. ^{19}F NMR spectrum of the catalytic conversion of **5g** into **6g** in THF-d_8. **# =** trifluorotoluene

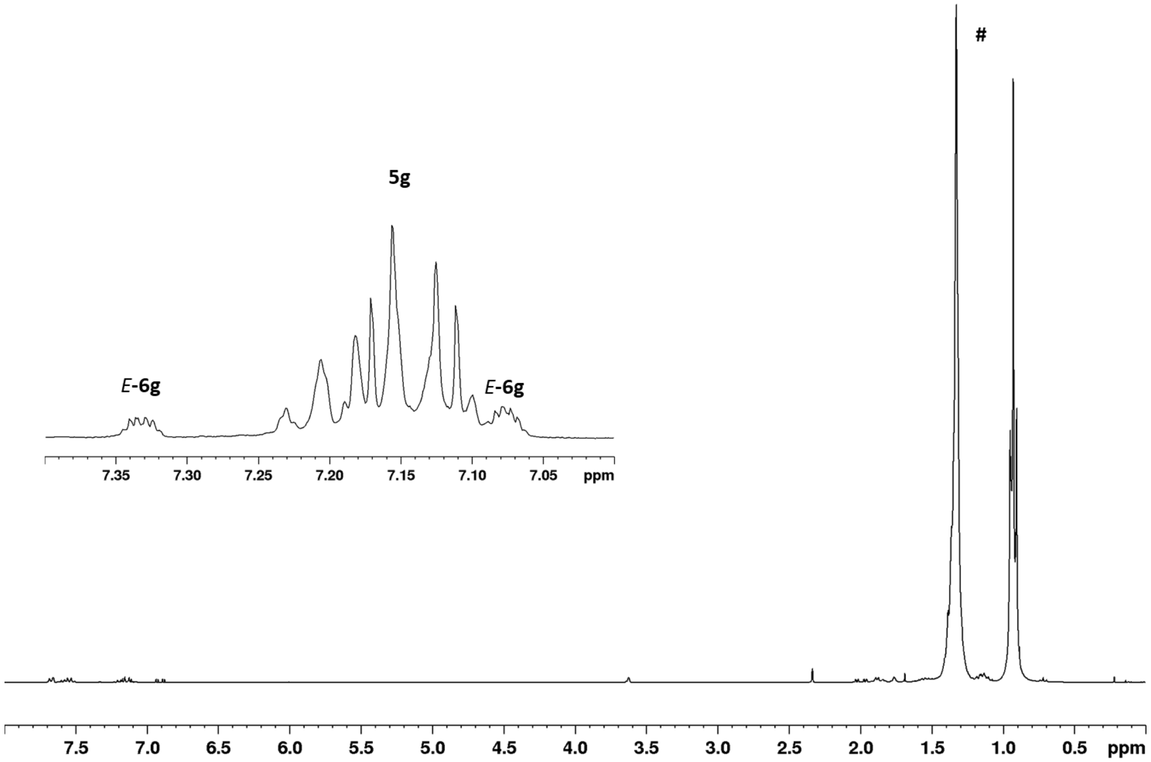

S21. ^1H NMR spectrum of the catalytic conversion of **5g** into **6g** in THF-d_8. **# =** heptanes

2. DFT calculations

2.1 Computational details for geometry optimization of the calculated complexes

Calculations were run using the Gaussian 16 (Revision A.03) program package.[10] Rhodium complexes were calculated using the BP86 functional. Rhodium was described with RECPs and the associated def2-SVP basis sets.[11] All the other atoms were described with def2-SVP basis sets. A Grimme D3 dispersion correction with Becke-Johnson damping was included.[12] Solvent effects have been taken into account using the SMD continuum solvent model,[13] employing toluene. All calculated structures were identified as minima (no negative eigenvalues).

2.2 Geometry optimization of complex 2

While different starting geometries were attempted in gas phase, minima were only found for tetragonal pyramidal structure where two possible isomers were identified based on the *cis*/*trans* isomerism of the alkenyl ligand.

Energy in Hartree (corrected for zero-point energy): -3581.117971 for *cis* isomer and -3581.115161 for *trans* isomer. Accordingly, *cis* structure at the alkenyl ligand is favored by 7.4 kJ/mol.

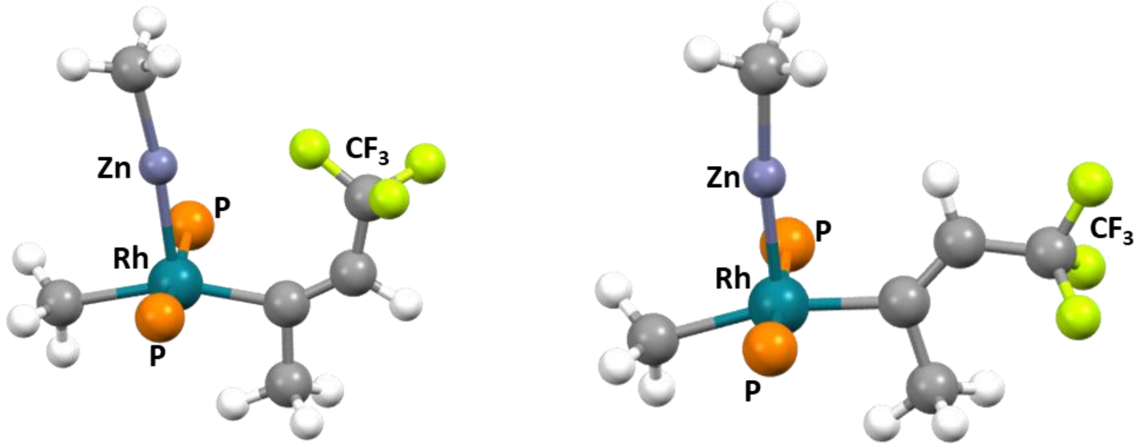

S22. DFT calculated geometry of the *Z* (left) and *E* (right) isomers of compound **2**. Ethyl groups of the phosphine ligands have been omitted for clarity.

Table S3. Cartesian coordinates of both optimized structures

***Z*-isomer**				***E*-isomer**			
Rh	0.03193	-0.58530	-0.56181	Rh	0.08997	-0.59408	-0.52764
P	-2.28528	-0.53256	-0.30534	P	-2.23561	-0.74641	-0.36921
C	3.04842	1.20746	-0.34226	C	3.03090	1.21270	0.21668
C	3.28692	-1.27070	-1.78812	C	3.27495	-0.56437	-2.03493
C	3.14883	-1.20957	1.16447	C	3.33002	-1.56537	0.76233
P	2.32947	-0.51385	-0.37156	P	2.38670	-0.43058	-0.39483
C	-3.15454	0.97595	-0.98820	C	-3.18523	0.80426	-0.81780
C	-4.50284	1.40275	-0.39988	C	-4.52622	1.08261	-0.13156
C	-2.93955	-0.61342	1.44256	C	-2.86067	-1.15614	1.34116
C	-2.48608	-1.83059	2.24780	C	-2.39700	-2.51509	1.86878
C	-3.13772	-1.96155	-1.15238	C	-3.02441	-2.05627	-1.43650
C	-4.66343	-2.05991	-1.09131	C	-4.54331	-2.23168	-1.37562
C	2.90155	-2.69828	-2.17764	C	2.85914	-1.72425	-2.93993
C	4.56376	1.37315	-0.21186	C	4.54495	1.42987	0.25953
H	-3.23379	0.78869	-2.08219	H	-3.29918	0.76334	-1.92360
H	-2.42023	1.80021	-0.88706	H	-2.49117	1.64284	-0.61178
H	-4.04781	-0.56429	1.40401	H	-3.96926	-1.10084	1.34717
H	-2.60232	0.32800	1.93049	H	-2.50375	-0.33052	1.99572
H	-2.83833	-2.78115	1.79756	H	-2.81533	-3.35213	1.27289
H	-2.87731	-1.78177	3.28532	H	-2.71473	-2.66001	2.92215
H	-1.37825	-1.88230	2.31066	H	-1.29074	-2.61372	1.83313
H	-2.66651	-2.87275	-0.72974	H	-2.50589	-3.00289	-1.18076

H	-2.78728	-1.91430	-2.20698	H	-2.69682	-1.81624	-2.47172
H	-5.04339	-2.06353	-0.04836	H	-4.90243	-2.43973	-0.34612
H	-5.00779	-3.00416	-1.56519	H	-4.85791	-3.08897	-2.00858
H	-5.15858	-1.22667	-1.62972	H	-5.08289	-1.33732	-1.74754
C	2.68428	-2.56201	1.70225	C	2.92345	-3.03650	0.83115
H	-4.40658	1.70903	0.66164	H	-4.39907	1.24272	0.95865
H	-5.27744	0.61217	-0.45371	H	-5.26951	0.27221	-0.26917
H	-4.89240	2.28401	-0.95313	H	-4.97196	2.01367	-0.54223
H	3.11489	-0.58157	-2.64436	H	3.07038	0.40458	-2.54202
H	4.36946	-1.20808	-1.54276	H	4.36742	-0.59166	-1.83148
H	2.52122	1.72652	0.48505	H	2.58189	1.33948	1.22569
H	2.67127	1.69657	-1.26356	H	2.53735	1.98581	-0.40658
H	4.95568	0.96250	0.74125	H	5.06051	0.70654	0.92413
H	4.82967	2.45137	-0.23315	H	4.77347	2.44594	0.64558
H	5.11559	0.88644	-1.04269	H	5.00783	1.35455	-0.74610
H	3.21701	-2.79757	2.64778	H	3.52026	-3.56064	1.60741
H	2.88308	-3.38781	0.99024	H	3.08858	-3.56291	-0.12997
H	1.59687	-2.56556	1.91721	H	1.85103	-3.15082	1.08968
H	4.24311	-1.22668	0.97005	H	4.40893	-1.47129	0.51179
H	2.98463	-0.42612	1.93914	H	3.20333	-1.09723	1.76509
C	0.03110	1.22636	-1.50354	C	-0.05434	1.44620	-0.53412
C	-0.01694	2.57126	-1.29852	C	-0.23163	2.39442	0.43261
C	-0.01257	3.30020	-0.00872	H	-0.29690	2.13230	1.50312
H	3.08361	-3.41739	-1.35330	H	3.06542	-2.71022	-2.47681
H	3.49824	-3.03650	-3.05125	H	3.41580	-1.68324	-3.90017
H	1.82959	-2.76929	-2.44817	H	1.77589	-1.68747	-3.17018
C	0.05354	0.76220	-2.95841	C	0.03912	1.83958	-2.00231
H	-0.03057	3.28732	-2.14690	C	-0.37351	3.85616	0.18516
C	-0.04142	-2.73247	-0.34053	F	0.65518	4.40168	-0.54671
Zn	0.19078	-0.10282	1.72070	F	-0.42935	4.56159	1.35067
C	0.39368	0.13583	3.66481	F	-1.51583	4.19831	-0.51561
H	0.99850	1.05873	-3.46622	C	0.14869	-2.68154	-1.08363
H	-0.78656	1.18925	-3.55026	Zn	0.29163	-0.66952	1.80093
H	-0.01846	-0.35046	-3.05505	C	0.48924	-0.54159	3.75578
H	-0.74824	-3.16287	0.40436	H	1.04601	2.24849	-2.23869
H	0.92206	-3.25286	-0.17153	H	-0.69963	2.61203	-2.30780
H	-0.40539	-3.07665	-1.34142	H	-0.10685	0.95662	-2.66988
H	0.69083	-0.81694	4.15352	H	-0.45853	-3.37482	-0.45566
H	-0.56120	0.47256	4.12357	H	1.14884	-3.15218	-1.16357
H	1.16632	0.90021	3.89798	H	-0.29461	-2.73856	-2.10917
F	-0.24118	2.53960	1.10740	H	0.90777	-1.47826	4.18332
F	-0.96579	4.28829	0.01561	H	-0.49236	-0.35236	4.24135
F	1.17718	3.95352	0.23894	H	1.17018	0.29508	4.02360

3. References

[1] D. D. Perrin, W. L. F. Armarego, *Purification of Laboratory Chemicals*, 3rd ed., Butterworth/Heinemann, London/Oxford, **1988**.

[2] P. Zhao, J. F. Hartwig, *Organometallics* **2008**, *27*, 4749-4757.

[3] M. Talavera, T. Braun, *Chem. Sci.* **2022**, *13*, 1130-1135.

[4] a) M. Talavera, C. N. von Hahmann, R. Müller, M. Ahrens, M. Kaupp, T. Braun, *Angew. Chem. Int. Ed.* **2019**, *58*, 10688-10692; b) T. Braun, D. Noveski, M. Ahijado, F. Wehmeier, *Dalton Trans.* **2007**, 3820-3825.

[5] A. Hernán-Gómez, S. A. Orr, M. Uzelac, A. R. Kennedy, S. Barroso, X. Jusseau, S. Lemaire, V. Farina, E. Hevia, *Angew. Chem. Int. Ed.* **2018**, *57*, 10630-10634.

[6] A. Hamieh, R. Dey, B. Nekoueishahraki, M. K. Samantaray, Y. Chen, E. Abou-Hamad, J.-M. Basset, *Chem. Commun.* **2017**, *53*, 7068-7071.

[7] P. H. M. Budzelaar, Version 4.1 ed., Adept Scientific plc, Letchworth, **2001**.

[8] M. Y. DeWolf, J. D. Baldeschwieler, *J. Mol. Spectrosc.* **1964**, *13*, 344-359.

[9] G. K. S. Prakash, H. S. Krishnan, P. V. Jog, A. P. Iyer, G. A. Olah, *Org. Lett.* **2012**, *14*, 1146-1149.

[10] M. J. Frisch, G. W. Trucks, H. B. Schlegel, G. E. Scuseria, M. A. Robb, J. R. Cheeseman, G. Scalmani, V. Barone, G. A. Petersson, H. Nakatsuji, X. Li, M. Caricato, A. V. Marenich, J. Bloino, B. G. Janesko, R. Gomperts, B. Mennucci, H. P. Hratchian, J. V. Ortiz, A. F. Izmaylov, J. L. Sonnenberg, D. Williams-Young, F. Ding, F. Lipparini, F. Egidi, J. Goings, B. Peng, A. Petrone, T. Henderson, D. Ranasinghe, V. G. Zakrzewski, J. Gao, N. Rega, G. Zheng, W. Liang, M. Hada, M. Ehara, K. Toyota, R. Fukuda, J. Hasegawa, M. Ishida, T. Nakajima, Y. Honda, O. Kitao, H. Nakai, T. Vreven, K. Throssell, J. E. P. J. A. Montgomery Jr., F. Ogliaro, M. J. Bearpark, J. J. Heyd, E. N. Brothers, K. N. Kudin, V. N. Staroverov, T. A. Keith, R. Kobayashi, J. Normand, K. Raghavachari, A. P. Rendell, J. C. Burant, S. S. Iyengar, J. Tomasi, M. Cossi, J. M. Millam, M. Klene, C. Adamo, R. Cammi, J. W. Ochterski, R. L. Martin, K. Morokuma, O. Farkas, J. B. Foresman, D. J. Fox, Revision A.03 ed., Gaussian, Inc., Wallingford CT, **2016**.

[11] a) D. Andrae, U. Häußermann, M. Dolg, H. Stoll, H. Preuß, *Theor. Chim. Acta* **1990**, *77*, 123-141; b) F. Weigend, R. Ahlrichs, *Phys. Chem. Chem. Phys.* **2005**, *7*, 3297-3305.

[12] a) S. Grimme, S. Ehrlich, L. Goerigk, *J. Comput. Chem.* **2011**, *32*, 1456-1465; b) S. Grimme, J. Antony, S. Ehrlich, H. Krieg, *The Journal of Chemical Physics* **2010**, *132*, 154104.

[13] A. V. Marenich, C. J. Cramer, D. G. Truhlar, *The Journal of Physical Chemistry B* **2009**, *113*, 6378-6396.
